# Supplementary material for: Desiderata for the development of next-generation electronic health record phenotype libraries
Source: Gigascience. 2021 Sep 11;10(9):giab059. doi: 10.1093/gigascience/giab059 (PMC8434766; doi:10.1093/gigascience/giab059)
Supplement: giab059_GIGA-D-21-00157_Revision_1 [file giab059_giga-d-21-00157_revision_1.pdf]

GigaScience

Desiderata for the development of next-generation electronic health record phenotype libraries

--Manuscript Draft--

**Manuscript Number:**	GIGA-D-21-00157R1
**Full Title:**	Desiderata for the development of next-generation electronic health record phenotype libraries
**Article Type:**	Review

Funding Information:

Health Data Research UK (NIWA1)	Prof Spiros Denaxas
Health Data Research UK (HDRUK/CFC/01)	Dr Andreas Karwath<br>Prof Georgios V Gkoutos
Innovative Medicines Initiative (116074)	Prof Spiros Denaxas
National Institute for Health Research	Dr Martin Chapman<br>Dr Andreas Karwath<br>Prof Georgios V Gkoutos<br>Dr Vasa Curcin
Nanocommons H2020 (731032)	Dr Andreas Karwath<br>Prof Georgios V Gkoutos
Medical Research Council (MR/S003991/1)	Dr Andreas Karwath
European Molecular Biology Laboratory	Dr Helen Parkinson
National Institute of General Medical Sciences (R01GM105688)	Mr Luke V Rasmussen<br>Ms Jennifer A Pacheco
National Human Genome Research Institute (U01HG011169)	Mr Luke V Rasmussen<br>Ms Jennifer A Pacheco

Abstract:

Background   High-quality phenotype definitions are desirable to enable the extraction of patient cohorts from large electronic health record (EHR) repositories, and are characterised by properties such as portability, reproducibility and validity. Phenotype libraries, where definitions are stored, have the potential to contribute significantly to the quality of the definitions they host. In this work, we present a set of desiderata for the design of a next-generation phenotype library that is able to ensure the quality of hosted definitions by combining the functionality currently offered by disparate tooling.

Methods   A group of researchers examined work to date on phenotype models, implementation and validation, as well as contemporary phenotype libraries developed as a part of their own phenomics communities. Existing phenotype frameworks were also examined. This work was translated and refined by all the authors into a set of best practices.

Results   We present 14 library desiderata that promote high-quality phenotype definitions, in the areas of modelling, logging, validation and sharing and warehousing.

Conclusions There are a number of choices to be made when constructing phenotype libraries. Our considerations distil the best practices in the field and include pointers towards their further development to support portable, reproducible, and clinically valid phenotype design. The provision of high-quality phenotype definitions enables EHR data to be more effectively used in medical domains.

Corresponding Author:

Martin Chapman
King's College London
London, UNITED KINGDOM

Corresponding Author Secondary Information:

Powered by Editorial Manager® and ProduXion Manager® from Aries Systems Corporation

**Corresponding Author's Institution:**	King's College London
**Corresponding Author's Secondary Institution:**	
**First Author:**	Martin Chapman
**First Author Secondary Information:**	
**Order of Authors:**	Martin Chapman
	Shahzad Mumtaz
	Luke V Rasmussen
	Andreas Karwath
	Georgios V Gkoutos
	Chuang Gao
	Dan Thayer
	Jennifer A Pacheco
	Helen Parkinson
	Rachel L Richesson
	Emily Jefferson
	Spiros Denaxas
	Vasa Curcin
**Order of Authors Secondary Information:**	
**Response to Reviewers:**	Department of Population Health Sciences<br>King's College London<br>London<br>SE1 1UL<br><br>July 15, 2021<br><br>Gigascience - Response to reviews<br><br>We would like to thank both reviewers for taking the time to produce excellent and in-depth reviews. Responses to the comments are provided below, many of which correspond to changes in the original article, and are highlighted in the version with tracked changes. We hope that we have been able to adequately address all the concerns.<br><br>* Reviewer 1<br><br>"In this work, the authors present a set of 13 desiderata to guide the development of future phenotype libraries.<br>The work presented here nicely rounds out current and established phenotyping efforts and established/outlines their suitability and components for a larger and broader definition of a phenotype library.<br>The relevant literature is well collected, and with the exception of newer developments (within the last 9 months) for the OHDSI phenotype library (https://data.ohdsi.org/PhenotypeLibrary/) and tools (https://pubmed.ncbi.nlm.nih.gov/31369862/) is highly relevant and up to date.<br>Each current tool is nicely analyzed and dissected by the authors to deliberate over the items that are included in the desiderata they propose."<br><br>> We thank the reviewer for their encouraging feedback, and agree that recent developments in the OHDSI network will indeed be a useful addition. We have now made changes in the text to recognise the initial deployment of the Gold Standard OHDSI Phenotype library (\`Background', Page 2, 9th paragraph) and the PheValuator tool (\`Automated multiple validation techniques', Page 8, 1st (full) paragraph).

Powered by Editorial Manager® and ProduXion Manager® from Aries Systems Corporation

"The figures and tables are well utilized and relevant, but a missing opportunity is a more comprehensive table that includes their 13 elements as columns and the current available libraries/tools as rows, with checkmarks as to which elements they provide in perspective to the 13 provided here."

> We agree that such a table would be useful, but do have some concerns about trying to draw comparisons between different libraries and tools under our desiderata in this manner.
For example, our desiderata focus on broad features and principles, which are often still under development in existing systems or exist in various forms that are not easily aligned.
We hope that our focus in this work will help advance the field to the stage where a meaningful direct comparison, such as the one suggested, can be made between different systems.

"One considerable concern is that the 13 desiderata feel like they are all proposed based on the authors' works (CALIBER and PhenoFlow), serving more of a way to fit these contributions to a broader context, than an impartial discussion about what phenotype libraries would need based on current literature.
Some changes in the language would greatly improve this, or the paper focus should be the phenotype library that the authors have built, versus the other approaches - which does not seem to be the way the manuscript is currently presented."

> We agree that a significant number of our desiderata are based upon the functionality offered by the tools and libraries developed within the authors' own phenomics communities.
In this form, the desiderata do indeed operate as `lessons learned', representing practices that have lead to the development of high-quality phenotype definitions and can thus inform the wider phenomics community.
We have clarified this at various points within the manuscript, including the abstract, introduction (Page 2, 5th paragraph) and methods (Page 3, 1st, 2nd and 3rd paragraphs) sections.

> To ensure that we are reflecting a broader perspective, our desiderata are further informed by our review of the functionality offered by tools outside of the authors' phenomics communities, such as those developed within the OHDSI network.
Thus, we would prefer to retain the concept of desiderata to allow ourselves the flexibility to also make reference to these externally developed tools, but the aforementioned additions to the manuscript make clear that the authors' own work contributes significantly towards the practices put forward.
The use of the term also gives us the flexibility to discuss our vision for future directions, albeit still grounded in concrete experiences.

"Other than this concern, this work is highly relevant and very useful for the communities involved in building phenotyping libraries."

> We thank the reviewer for all their positive remarks.

* Review 2

"High-quality phenotype definitions are desirable for clinical research.
A phenotype library of portable, reproducible and validated phenotyping definitions will be valuable for the research community.
The authors examined the work phenotyping models, implementation and validation, and summarized several desiderata for best practices in this review.
Some points mentioned in the paper were similar to the previous report cited (https://academic.oup.com/jamia/article/22/6/1220/2357938)."

> We thank the reviewer for their in-depth summary of the work.

"My primary concern regarding this piece of work is the phenotyping scope.
The discussion and thoughts fit well for most rule-based phenotype definitions.
However, more and more phenotyping research moves forward to either machine-

	learning-based or high-throughput approaches (e.g., PheMAP and PheNorm).<br>Therefore, it is necessary to add discussions on these approaches.<br>In addition, NLP algorithms could be vastly complicated.<br>Therefore, it is essential to add more discussions regarding the complexity beyond NLP languages and packages."<br><br>> We agree that an increased focus on machine-learning-based and natural language processing-based/high-throughput approaches is required.<br>We have added additional recognition for these approaches, alongside traditional rule-based approaches, at various points in the article, including our introduction to phenotyping (Page 2, 1st paragraph), our closing discussion (Page 10, 3rd (full) paragraph) and within the desiderata themselves (Page 5, 2nd, 3rd and 4th paragraphs).<br><br>> In the latter case, we have developed an additional desideratum within the `models' section -- `Support Natural Language Processing-based and Machine Learning-based definitions' -- which significantly expands upon our comments about the importance of abstract models in representing a wider range of definition types, including ML and NLP approaches.<br>Specifically, we have expanded our discussion of NLP-based phenotypes, discussing complex processes such as those associated with the derivation of the PheMap knowledge base.<br>In addition, we have expanded our discussion on machine learning-based approaches, to provide more details on the processes for deriving probabilistic phenotypes, such as the operation of the PheNorm algorithm.<br><br>* Editor<br><br>"- reviewer 1 points out that ` the 13 desiderata feel like they are all proposed based on the authors' works (CALIBER and PhenoFlow)'. For a narrative review article such as this, it is not a problem if it presents `lessons learned' based on the authors' own work, but I agree with the reviewer that this should be reflected in the language of the article.<br>- reviewer 2 feels a section on machine-learning-based and high-throughput approaches is needed."<br><br>> We hope that, in our response to the individual reviewers, we have been able to address these concerns.<br><br>"In addition, I recommend to improve the title of the article, to make it clear it's about phenotype libraries in a clinical context. GigaScience is a multidisciplinary journal and I think it would be wise to make it clear in the title that this review is about phenotypes in the context of health records."<br><br>> We have altered the title accordingly.<br><br><br>Best regards,<br><br>Martin Chapman
**Additional Information:**	
**Question**	**Response**
Are you submitting this manuscript to a special series or article collection?	No
**Experimental design and statistics**<br><br><br>Full details of the experimental design and statistical methods used should be given in the Methods section, as detailed in our Minimum Standards Reporting Checklist.	Yes

Information essential to interpreting the data presented should be made available in the figure legends.<br><br>Have you included all the information requested in your manuscript?	
**Resources**<br><br>A description of all resources used, including antibodies, cell lines, animals and software tools, with enough information to allow them to be uniquely identified, should be included in the Methods section. Authors are strongly encouraged to cite Research Resource Identifiers (RRIDs) for antibodies, model organisms and tools, where possible.<br><br>Have you included the information requested as detailed in our Minimum Standards Reporting Checklist?	Yes
**Availability of data and materials**<br><br>All datasets and code on which the conclusions of the paper rely must be either included in your submission or deposited in publicly available repositories (where available and ethically appropriate), referencing such data using a unique identifier in the references and in the "Availability of Data and Materials" section of your manuscript.<br><br>Have you have met the above requirement as detailed in our Minimum Standards Reporting Checklist?	Yes

Powered by Editorial Manager® and ProduXion Manager® from Aries Systems Corporation

Click here to access/download;LaTeX - Other (clo, cls, sty etc.);tracked-changes.pdf

OXFORD (GIGA)^n SCIENCE

GigaScience, 0000, 1–13

doi: xx.xxxx/xxxx
Manuscript in Preparation
Review

REVIEW

Desiderata for the development of next-generation electronic health record phenotype libraries

Martin Chapman [0000-0002-5242-9701][1,*], Shahzad Mumtaz [0000-0003-2606-2405][2], Luke V. Rasmussen [0000-0002-4497-8049][3], Andreas Karwath [0000-0002-6942-3760][4], Georgios V. Gkoutos [0000-0002-2061-091X][4], Chuang Gao [0000-0002-6456-0301][2], Dan Thayer [0000-0003-1847-4362][5], Jennifer A. Pacheco [0000-0001-8021-5818][3], Helen Parkinson [0000-0003-3035-4195][6], Rachel L. Richesson [0000-0003-0279-7036][7], Emily Jefferson [0000-0003-2992-7582][2], Spiros Denaxas [0000-0001-9612-7791][8] and Vasa Curcin [0000-0002-8308-2886][1]

[1]King's College London, London, UK and [2]Health Informatics Centre (HIC), University of Dundee, Dundee, UK and [3]Northwestern University Feinberg School of Medicine, Chicago, Illinois, USA and [4]University of Birmingham, Birmingham, UK and [5]SAIL Databank, Swansea, UK and [6]European Molecular Biology Laboratory - European Bioinformatics Institute (EMBL-EBI), Hinxton, UK and [7]University of Michigan Medical School, Michigan, USA and [8]University College London, London, UK

* Corresponding Author. Email: martin.chapman@kcl.ac.uk

Abstract

Background High-quality phenotype definitions are desirable to enable the extraction of patient cohorts from large electronic health record (EHR) repositories, and are characterised by properties such as portability, reproducibility and validity. Phenotype libraries, where definitions are stored, have the potential to contribute significantly to the quality of the definitions they host. In this work, we present a set of desiderata for the design of a next-generation phenotype library that is able to ensure the quality of hosted definitions by combining the functionality currently offered by disparate tooling. **Methods** A group of researchers examined work to date on phenotype models, implementation and validation, as well as contemporary phenotype libraries developed as a part of their own phenomics communities. Existing phenotype frameworks were also examined. This work was translated and refined by all the authors into a set of best practices. **Results** We present ~~13~~ 14 library desiderata that promote high-quality phenotype definitions, in the areas of modelling, logging, validation and sharing and warehousing. **Conclusions** There are a number of choices to be made when constructing phenotype libraries. Our considerations distil the best practices in the field and include pointers towards their further development to support portable, reproducible, and clinically valid phenotype design. The provision of high-quality phenotype definitions enables EHR data to be more effectively used in medical domains.

Key words: Electronic health records; EHR-based phenotyping; computable phenotype; phenotype library

Compiled on: July 15, 2021.
Draft manuscript prepared by the author.

Key Points

- Portable, reproducible and clinically valid phenotype definitions have the potential to unlock health data repositories for wider and more effective use.
- To ensure definitions are of high quality, associated tools should be supported directly through the libraries where phenotype definitions are hosted.
- ~~13~~ 14 desiderata are presented to guide the development of future phenotype libraries, and to ensure phenotype definitions are of a sufficient quality to enable the effective use of medical data in research and in healthcare provision.

Introduction

As a result of digitisation of health systems world-wide, electronic health record (EHR) data repositories have emerged as the main source of data for medical cohort research studies. To extract these cohorts, there is an increasing reliance on EHR-based *phenotype* definitions (also referred to as phenotyping algorithms), which identify individuals that exhibit certain phenotypic traits, such as the same diseases, characteristics, or set of comorbidities. These definitions can be represented in many forms, including narrative descriptions, pseudo-code, or, in some cases, may already be directly executable. Conceptually, they may vary from simple code lists, via rule-based algorithms to more involved machine learning (ML) tasks and high-throughput approaches using natural language processing (NLP).

While traditional big data techniques can successfully address the scale of the EHR data available, the effectiveness of phenotype definitions is impacted by a range of other syntactic and semantic issues, including variations in the way data is structured and the coding systems used.

To overcome these issue and enable effective cohort extraction, a phenotype definition must exhibit certain properties. It must be *reproducible* allowing for accurate (re)implementation, irrespective of the idiosyncrasies of the dataset against which the definition was originally developed; *portable*, allowing for straightforward implementation, irrespective of the structure of the target dataset; and *valid*, effectively capturing the disease or condition modelled. A definition that exhibits all of these properties we refer to as ~~*high-quality*~~*high quality*.

To ensure high-quality phenotype definitions, support should be provided to the authoring, implementation, validation and dissemination processes of a phenotype's *lifecycle*. While such support is currently available, it is often sporadic and inconsistent as it is delivered via a wide range of different tools. Instead, building on the work of Richesson et. al [1], we propose that the functionality provided by these tools should instead be provided centrally, through the phenotype *libraries* where definitions are hosted. For example, libraries should enable phenotypes to be developed according to some set of standard models, and track the evolution of definitions under these models, so as to ensure hosted definitions are clearer to understand and thus have the potential to be more reproducible. Moreover, libraries should assist in the derivation of directly computable phenotype definitions, through the provision of implementation tooling, to improve portability by enabling the execution of phenotypes in local use cases. Similarly, libraries should directly validate the definitions they host, through, for example, automated comparisons with gold standards.

To this end, in this work we contribute a number of desiderata for the development of phenotype libraries, which not only ensure that definitions are accessible, but also maximise the quality of the phenotypes they contain by supporting all parts of the definition lifecycle. These desiderata are based on both the lessons learned during the development of contemporary libraries within the authors' own phenomics communities, as well as a review of the functionality currently offered by phenotype tooling, which represent practices that have lead to the development of high-quality phenotype definitions. By providing access to high-quality definitions, phenotype libraries enable both efficient and accurate use of EHR data for activities such as medical research, decision support and clinical trial recruitment.

Background

Human phenomics is the study of human phenotypes, and includes the science and practice of defining observable medical phenomena that indicate phenotypes to advance research and personalised care. The concept of a phenotype originated as a complement to the genotype, and a phenome was defined as a complete set of an individual's inheritable characteristics. Rather than describing someone's genetic information, a phenome captures all the observable properties (phenotypes) that result from the interaction of their genetic make-up and environmental factors, including their demographic information, such as height or eye color, and medical histories.

With the emergence of large-scale EHR data repositories, the term phenotype has evolved to denote traits shared by groups of patients, such as a disease or condition that a cohort, or set of individuals, has. This may also include other complex combinations of traits, exposures, or outcomes, including comorbidities, polypharmacy, and demographic data. Defining these phenotypes, and validating them to ensure their accuracy and generalisability, is the process known as *phenotyping*, with *EHR-based phenotyping* relying primarily on data in the EHR. *Computational phenotyping* (also known as *deep phenotyping*) uses either supervised machine learning techniques to discover new members of a priorly defined cohort, or unsupervised techniques to discover entirely new phenotypes and investigate their properties.

EHR data repositories bring with them a very specific set of data challenges in terms of managing syntactic and semantic complexity, which act as a barrier to studies that need to utilise patient information from across multiple data sources and for the needs of different studies. For example, by the nature of healthcare delivery and how EHRs are used to document, a patient who has been diagnosed with diabetes mellitus may be represented slightly differently in two EHR systems, and will almost certainly be represented differently in EHRs for different countries.

Phenotype *libraries* – where definitions can be uploaded, stored, indexed, retrieved, and downloaded by users – provide a logical place in which to ensure that definitions are of a suitable quality to overcome many of the issues associated with extracting cohorts from complex EHR datasets. This is accentuated by the fact that the development of phenotype libraries is a rapidly growing area~~, with several currently under, or planned for, development. Examples include the VAPheLib — which aims to collect, store and make available 1000 curated phenotype definitions for the clinical operations research community by the end of 2021 — and the~~ . Of particular note is the Observational Health Data Sciences and

Informatics (OHDSI) Gold Standard Phenotype Library, which aims to support OHDSI community members in finding, evaluating and utilising cohort definitions that are validated by the research community. An initial version of the library is currently available, alongside a wider set of requirements to guide its future development [3]. Other libraries planned for development include the VAPheLib [4], which aims to collect, store and make available 1000 curated phenotype definitions for the clinical operations research community by the end of 2021. Phenotype libraries are also being developed as a part of wider phenotype frameworks. Alongside Richesson's reusable phenotype definition framework sit initiatives such as the *phenotyping pipeline* (PheP), which aims to extract, structure and normalise phenotypes from EHR data collected across participating sites [5].

Methods

To determine the functionality that should be provided by a next-generation phenotype library, a team of international researchers ~~from leading phenomics communities~~ – comprising Health Data Research UK (HDR UK) Phenomics theme members and US researchers from the Mobilizing Computable Biomedical Knowledge (MCBK) and Phenotype Execution and Modelling Architecture (PhEMA) communities – first examined a range of tools supporting different parts of the definition lifecycle, ~~including~~ which were developed within their respective phenomics communities. This was enriched with a wider review of the literature via Web of Science (WoS) [6] and the grey literature via Google to identify third-party projects that have developed phenotype tooling, or are planning its development, and future trends. Our decision to include the grey literature was informed by our a priori knowledge of tools under development that have not yet published peer-review articles. The tools reviewed included those that support authoring (e.g., modelling using the Quality Data Model (QDM) logic [7], the Clinical Quality Language (CQL) [8], and use of the Observational Medical Outcomes Partnership (OMOP) Common Data Model (CDM) [9] and associated tooling such as OHDSI's Automated PHenotype Routine for Observational Definition, Identification, Training and Evaluation (APHRODITE) [17]), implementation (e.g., definition translators [11]) and validation (e.g., electronic phenotyping validation [12])~~tools.~~ Common functionality provided by the tools identified – representing opportunities for new phenotype library functionality – was extracted and summarised.

~~A purposive sample of existing library initiatives~~ In addition, the authors examined existing libraries from within their own communities – including the Phenotype Knowledge Base (PheKB) [13], CALIBER [14], Phenoflow [15] ~~,~~ and the Concept Library [16] ~~and OHDSI's Automated PHenotype Routine for Observational Definition, Identification, Training and Evaluation (APHRODITE) computable phenotype architecture – were also examined to determine existing functionality and future trends. This was enriched with a review of the literature via Web of Science (WoS) and the grey literature via Google to identify projects that have developed or planned development of a phenotype library. Our decision to include the grey literature was informed by our a prioriknowledge of systems under development that have not yet published peer-review articles.~~ to identify instances of functionality currently supporting the phenotype definition lifecycle. Common functionality provided by ~~both the tooling and existing libraries were~~ these libraries – which has been shown to result in reproducible, portable and valid phenotype definitions, and thus represent best practice – was also extracted and summarised. ~~These~~

Both of these summaries were translated to a draft set of desiderata via discussion amongst a subset of the authors [MC, SM, EJ, SD, VC]. All authors participated in an asynchronous iterative review process to critique, consolidate, refine, and define the final set of desiderata. The desiderata were further classified into logical categories.

Desiderata

In total, the authors arrived at a finalised collection of ~~13~~ 14 desiderata, which are organised across the following sections into five categories: modelling, logging, implementation, validation and sharing and warehousing. Figure 1 shows how the desiderata presented promote the design of a phenotype library that supports all parts of the phenotype definition lifecycle.

Modelling

Phenotype models govern the structure and syntax of phenotype definitions. For example, phenotype definitions are ~~commonly~~ traditionally rule-based, meaning that they are comprised of individual logical statements that each evaluate to a boolean value, typically by relating *data elements* (with associated *values*) – such as the presence of a particular set of ICD-10 codes or a particular lab result – to each other. The set of operators available to an author when connecting data elements (e.g. logical connectives such as conjunction and disjunction) would be established within a phenotype definition model. A model may dictate that a phenotype be represented in an unstructured, semi-structured, structured, or executable manner [18]. A summary of different phenotype definition formats, governed by phenotype models, is given in Table 1.

Implementing a phenotype definition involves translating the abstract definition (if unstructured or semi-structured) into an executable form that can be directly run against a patient dataset in order to derive the cohort exhibiting the defined phenotype. Typically this requires the logic of the definition to be realised in a programming language, such as translating abstract conditional clauses into a set of tangible Python conditional statements. We refer to these implementations as *computable phenotypes*. For a definition to be reproducible, it must be realised in a formal structure that can be accurately interpreted and implemented. Given the potential for human error in translating from an unstructured narrative to something computable, formal phenotype models provide such a structure.

Phenotype models are also key in ensuring semantic interoperability between definitions. That is, while the development of phenotype definitions can involve deriving a curated, canonical set of phenotype definitions containing 'definitive' versions for each disease of condition being modelled for a particular domain (e.g. a national stroke body may want to maintain their set of stroke phenotyping algorithms), more often than not, it is perfectly valid to have overlapping phenotype definitions for different uses. For example, an eligibility criteria for a clinical trial may differ from a rule that triggers a decision support tool in an EHR system, and both would differ from a definition used in a population health study, even if all three nominally refer to same disease [19]. Internationally, definitions for the same disease may also differ [20]. While this overlap is permissible, different definitions for the same condition must still be compatible, enabling, for example, their relative functionality to be compared. The adoption of a phenotype model enables such compatibility.

Given these benefits, a phenotype library should adopt a formal phenotype model to control the structure of hosted definitions. To ensure the use of such a model, a library can offer a graphical authoring environment – in the same way that tools such as the Phenotype Execution and Modelling architecture (PhEMA) Authoring Tool (PhAT) do [7] – through which new definitions can be au-

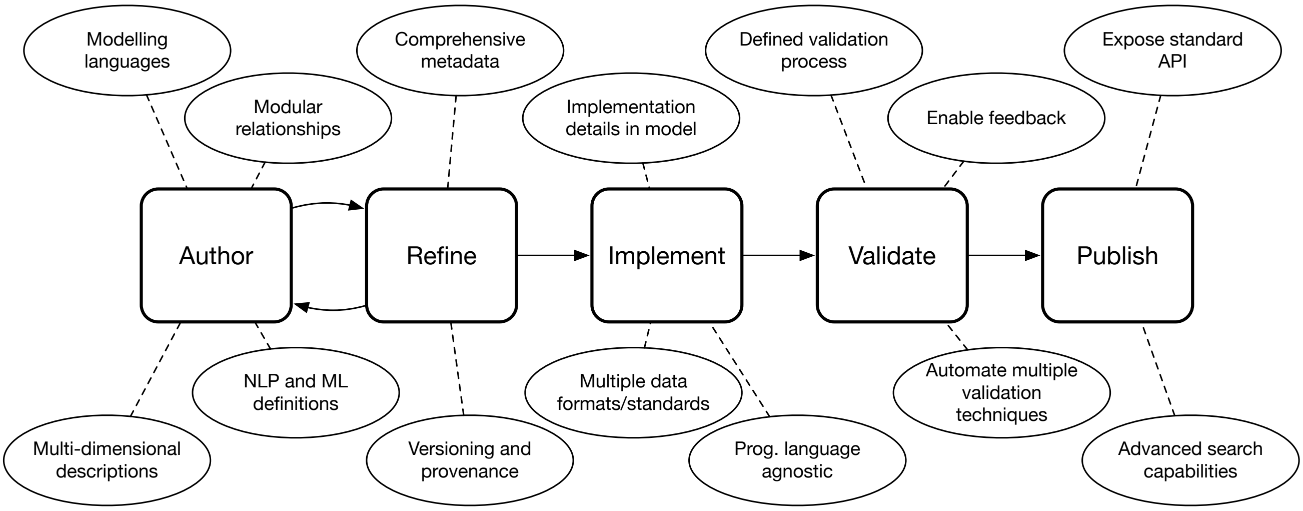

Figure 1. The stages of the phenotype definition lifecycle supported by a next-generation phenotype library.

Table 1. Phenotype definition formats

Format	Description	Example	Category
Code list	A set of codes that must exist in a patient's health record in order to include them within a phenotype cohort	COVID-19 ICD-10 code *U07.1*	Rule-based
Simple data elements	Formalising the relationship between code-based *data elements* using *logical connectives*	COVID-19 ICD-10 code *U07.1* AND ICD-11 code *RA01.0*	Rule-based
Complex data elements	Formalising the relationship between complex data elements, such as those derived via NLP.	Patient's blood pressure reading > 140 OR patient notes contain 'high BP'	Rule-based
Temporal	Prefix rules with temporal qualifiers	Albumin levels increased by 25% over 6 hours, high blood pressure reading has to occur during hospitalisation.	Rule-based
Trained classifier	Use rule-based definitions as the basis for constructing a classifier for future (or additional) cohorts	A k-fold cross validated classifier capable of identifying COVID-19 patients	Probabilistic

thored. Similarly, existing definitions can be automatically checked for their adherence to the chosen model when uploaded.

Desiderata relating to the adoption of a phenotype model by a library are listed in the following sections. We view these desiderata as complementary to the well-established desiderata for phenotype definition model development put forward by Mo et al. [21].

Support modelling languages

The phenotype definition model adopted by a library should be supported by a (non-executable) high-level modelling language that dictates the syntax available to an author when defining the logic of a phenotype. A computable form of the definition can then be realised for execution in a local use case. When selecting or developing a definition model, the temptation may be to select a lower-level, executable programming language, in an attempt to expedite local implementation. For example, one could argue that a language such as Python is sufficient for simultaneously defining phenotypes and realising them computationally. However, we would argue that using such a language as a means to express the logic of a definition ties the definition to general purpose, low-level language constructs, reducing clarity, and thus reproducibility. This conclusion is supported by work such as [22], which found openEHR an overly restrictive standard when attempting to express phenotype definitions in a form that can be directly executed. An example of a phenotype definition realised in an executable language (Python) is given in Figure 2.

In contrast, the syntax of ~~higher level~~ higher-level modelling languages, while still precise, is often clearer, as well as often being domain specific. For example PhEMA's PhAT allows users to define ~~phentoypes~~ phenotypes using the high-level, domain-specific

```
valueset={}
valueset[``Acute Pharyngitis"] = ``2.16.840...1011"
valueset[``Acute Tonsilitis"] = ``2.16.840...1012"

def Pharyngitis():
    conditionA = valueset[``Acute Pharyngitis"]
    conditionB = valueset[``Acute Tonsilitis"]
    return conditionA + `` " + conditionB;
```

```
valueset ``Acute Pharyngitis": ``2.16.840...1011"
valueset ``Acute Tonsilitis: ``2.16.840...1012"

define Pharyngitis:
    [Condition: ``Acute Pharyngitis"] union
       [Condition: ``Acute Tonsilitis"]
```

Figure 2. Python (executable) vs. CQL (modelling) [23] representation of Pharyngitis phenotype.

syntax associated with the Quality Data Model's (QDM) logic expressions (now capable of working instead with the Clinical Quality Language (CQL) [8]). Both QDM and CQL make particular provision for the representation of temporal information, such as the (sequential) relationship between events, or between events and defined measurement periods. A further example of a modelling language is OHDSI's cohort definition syntax, which although tied directly to the OMOP CDM, is also high-level and domain specific, allowing for significant clarity when interpreting existing definitions [9]. Like QDM/CQL, this syntax also makes provision for temporal elements (e.g. associating patient observations to an elapsed time period), but looks more holistically at the cohort relating to the phenotype being defined, through, for example, the use of ~~defined~~ specified inclusion and exclusion criteria. As a final example, *Phenoflow*'s

workflow-based model relies on a categorised set of steps to express phenotype definitions, with the same benefits [15]. An example of a phenotype realised in a higher level modelling language (CQL) is also given in Figure 2 for comparison.

~~In encouraging phenotype definition models to be built around modelling languages, there is also the potential to support the definition~~ It is also important to note that the use of a modelling language as the basis for a phenotype model does not preclude the utility or use of higher-level, (more) human-readable representations such as flowcharts. In fact, modelling languages typically connect well with such representations. For example, flowcharts can be directly generated from Phenoflow's workflow model, QDM is linked to a graphical HTML layer and OHDSI cohorts can be viewed graphically using the *ATLAS* cohort editor.

Support Natural Language Processing-based and Machine Learning-based definitions

The modelling language selected to form the basis of a phenotype definition model should also support the representation of a wider range of definition types (Table 1). That is, ~~at a higher level one is~~ under a definition model, one should be able to express not only standard rule-based definitions, but also more complex definitions based on Machine Learning (ML) and Natural Language Processing (NLP) ~~and Machine Learning (ML)~~ techniques. These techniques are ~~increasingly being used to either derive, or form a part of, phenotype definitions,~~ becoming increasingly prevalent, particularly in those situations where the datasets against which the implemented definition is to be executed ~~against~~ are of varying completeness ~~. For example, through a modelling language, an author should be able to formally express the synonyms of a given medical term, with a view to these being used as the basis for processing free-text from a medical record in a computable form in the absence of consistent record coding. Expressing the use of NLP in an executable language would likely require~~ or lack consistent record coding. The use of modelling languages to represent these types of definitions is also important for reproducibility, as the use of an abstract representation reduces the potential for references to implemented libraries, ~~which would reduce portability. Similarly, in the case of ML~~ commonly used by NLP and ML techniques.

Critically, in order to sufficiently represent both ML and NLP-based phenotypes, a modelling language ~~should support the~~ must be able to represent not only static information (as in rule-based phenotypes) but also complex processes. For example, in the case of ML, a definition may consist of a static, high-level specification of a trained patient classifier (via the provision of values such as feature coefficients), or ~~a~~ may be a more complex description of the workflow used to ~~derive a classifier~~ train a classifier for a given condition, with a view to the classifier being re-implemented in new use cases, or training a new model in new use cases, respectively~~. Once again, at a lower level, this would likely result in references to implemented libraries, reducing portability.The abstract~~ . The workflow used to train a classifier may involve the identification of cases using the presence of certain keywords within an EHR [24] or, as in the case of the *PheNorm* framework, may involve additional steps, such as *normalisation* (to factor in number of encounters when looking at the significance of a larger number of keywords or codes) and *denoising* (to look at the wider context of a keyword or code count, e.g. competing diagnoses) [25]. The high-level definition of machine learning-based, or probabilistic, phenotypes ~~is something~~ in this way is supported in the OHDSI's Automated PHenotype Routine for Observational Definition, Identification, Training and Evaluation (APHRODITE) computable phenotype ~~architecture~~framework, which, although also linked to the OMOP CDM, offers a level of abstraction at which ~~rules can be fed into the construction of a classifier, and lower level code generated accordingly~~ trained classifiers can be represented and ported between sites, or a defined workflow can be used to construct site-specific classifiers, when used in an executable form [26]. ~~Similarly, languages like CQL have the potential to link to *external tooling*, for the purposes of expressing NLP and ML functionality.~~

~~It is also important to note that the use of a modelling language as the basis for a phenotype model does not preclude the utility or use of higher-level, (more) human-readable representations such as flowcharts. In fact, modelling languages typically connect well with such representations. For example, flowcharts can be directly generated from Phenoflow's workflow model , QDM is linked to a graphical HTML layer and OHDSI cohorts can be viewed graphically using the *ATLAS* cohort editor~~In the case of NLP, a definition may consist of a simple list of keywords relating to a given medical concept, or a set of regular expressions (not tied to any specific programming language), with a view to these being used as the basis for identifying conditions from free-text in a medical record, when realised in a computable form. However, like ML, NLP-based phenotype definitions are also often associated with complex processes, especially when used to conduct *high-throughput* phenotyping. For example, a *PheMap* phenotype definition consists of a set of linked concepts, the presence of which in a patient's EHR are used to determine the probability of them having the condition represented [27]. The association of a phenotype with different concepts is defined within the PheMap knowledge base, which is constructed based on a process that uses a specific set of NLP tools to derive these associations based on the content of various text-based resources. Therefore, it is also important to represent this process as a part of any definition, especially if the knowledge base needs to be reconstructed within different domains. In instances such as these, it may be important for the definition model used to include guidance on the use of specific tools, but it must do so in a manner that retains clarity and generalisability, thus balancing reproducibility requirements. Modelling languages like CQL have the potential to link to *external tooling*, for the purposes of effectively capturing NLP and ML processes such as these.

Support multi-dimensional descriptions

A significant hurdle in porting a phenotype definition from one setting (institution or dataset) to another – a process we refer to as localisation – is understanding its structure and semantics in order to derive a local computable form, or modify an existing one. Complex rules and the use of idiomatic clinical terminology, although often necessary components of a definition, are both barriers to this understanding, and thus reproducibility. To address this issue, a phenotype definition model should allow an author to express the same logic of a phenotype at different levels of technical complexity. This approach aims to communicate supplementary information alongside the provision of the core definition logic. For example, the workflow-based Phenoflow model allows an author to use the technical terminology and rules required to express a phenotype definition, but then also requires an author to provide longer definitions of this functionality to improve clarity, and to also classify each unit of functionality under a given ontology, enabling a high-level understanding of the functionality to always be accessible. In other modelling languages like CQL, such information can be communicated using constructs such as inline comments.

Logging

The development of a phenotype definition is an incremental process. Capturing and communicating this process is key in ensuring a definition can be accurately interpreted and is thus reproducible. ~~Morover~~Moreover, this information strengthens the trustworthiness of a phenotype and thus its potential applications. Therefore,

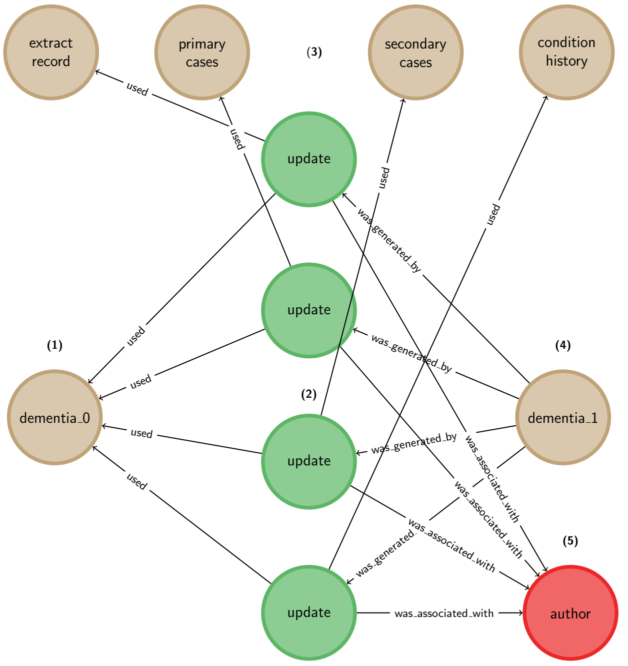

Figure 3. An example data provenance trace showing an update to a dementia phenotype, using the W3C PROV standard. The initial version of the phenotype (1) is updated by four edit activities (2), each of which modifies a component of the definition (e.g. record extract logic, diagnostic codes, previous history) (3), in order to generate a new version (4), and the process is linked with the author making these edits (5).

phenotype libraries should provide a mechanism for logging the evolution of a phenotype definition.

Support versioning and data provenance

One way in which a phenotype can evolve is through a series of iterative refinements. SAIL ~~databank~~Databank's Concept Library stores phenotypes as sets of codes, with a view to making these phenotypes available in different studies and use cases [16]. The concept library, as the name suggests, focuses on a model under which phenotypes are collections of grouped medical *concepts* or *working sets*. The Concept Library records and communicates the evolution of a phenotype definition using methods akin to standard version control, logging the state of a phenotype after each revision, and thus provides an overview of the definition's progression. This versioning process often relies on attributing a universally unique identifier (UUID) to each definition, and each subsequent revision of that definition. Such an identifier might simply be incremental, or convey some details of the phenotype itself. It should also be independent of other identifiers, in order to maximise clarity [28]. For example, within APHRODITE a UUID is ~~generated~~ derived by committing (each version of) a generated definition to a GitHub repository and extracting the unique commit hash value, in accordance with the FAIR (Findable, Accessible, Interoperable and Reusable) principles [29].

A more comprehensive way to capture the evolution of a definition – and thus contribute to its reproducibility – is to deploy formal *data provenance capture* tools to capture richer, real-time information about the evolution of an entity. This might include information about updates to the structure of a definition, or details of how that definition was validated. It might also include information about how the definition was derived if, for example, the definition is a trained model. An example of one such tool is the *data provenance template server* [30], which allows for the specification of abstract templates, based on the *W3C PROV* standard [31], while eliminating the complexity of dealing with low-level provenance constructs.

Using provenance tools, a trace is automatically constructed that can be *queried* in order to answer a range of questions, such as which clinical codes were used to support a definition at a given time. The Phenoflow library is integrated with the provenance template server, enabling the evolution of the definitions it hosts to be tracked over time [32]. A fragment of provenance constructed in this manner is shown in Figure 3.

Support modular relationships between phenotypes

Another way in which phenotype definitions evolve is through their reuse in constructing new definitions. For example, a phenotype may, either in part or entirely, be defined by other self-contained phenotypes. For example, bipolar disorder is (in part) defined by both substance and alcohol abuse, two phenotypes in their own right [33]. In this way, existing phenotypes become the building blocks for new phenotypes. Much like a version history, it is thus important to capture and communicate this information upon implementation, to provide detailed insight into the formulation of the definition. As such, a phenotype library should log the relationship between different definitions and, if authoring capabilities are supported, a library should allow new definitions to be constructed based upon existing ones. This is similar to the approach taken by the Concept Library, which relates concepts to each other in order to create phenotype definitions, and by Finngen's *Risteys* platform, which relates phenotypes temporally, listing those phenotypes that a patient is likely to exhibit either before or after exhibiting another (e.g. the onset of depression after exhibiting bipolar disorder) [34]. Establishing this relationship further contributes to the provenance of a phenotype, the precision of its definition, and, consequently, its reproducibility.

Conversely, *sub-phenotypes* may be computationally derived from existing phenotypes by clustering of those features (e.g. demographic, diagnosis, medication, etc.) identified, by a trained classifier, to be key attributes of those patients exhibiting the parent phenotype [35]. Such a relationship should also be logged by a phenotype library, to establish the evolution of a definition, and track changes and dependencies across phenotype definitions.

Implementation

Our initial desiderata determined that phenotype definitions should not themselves be executable. While important for reproducibility, this raises natural issues around the complexity of realising a phenotype defined using a modelling language computationally for individual use cases, something that negatively impacts portability. This issue can be addressed by meeting several requirements, which are explored in the following sections.

Communicate implementation information in the model

One way in which implementation can be supported is through the definition itself, by communicating information pertinent to its computable realisation. To do this, one might select a phenotype definition model based on a modelling language that allows an author to express additional information at different levels of *abstraction*. For example, the Phenoflow model frames the traditional (rule-based) logic of a phenotype definition as an *abstract* layer, and allows an author to complement this layer with additional layers, each of which gradually communicates more implementation information: a *functional* layer, introducing the concept of data types, and a *computational* layer, expressing details such as target execution environments. The fact that these layers sit alongside the traditional, abstract logic layer, allows for more concrete implementation to be expressed without impacting portability.

The abstract layer of the Phenoflow model is split into individual modules, each of which represents a distinct unit of functionality, and which collectively define the process required for deriving a pa-

tient cohort from a set of health records. Each module in the abstract layer has an equivalent module in both the functional and computational layers, ensuring a correspondence between each level of representation within the model. However, these modules also provide another means by which implementation information can be communicated through a definition model, in that they provide a clear template for development; each module represents a single unit of functionality that must be implemented by a developer when realising the computable form. This reduces the implementation burden on developers, and thus improves portability. Modelling languages like CQL, which support the definition of individual functions as a part of an abstract layer, offer similar benefits.

Support tooling that provides multiple programming language implementations

Phenotype *implementation tooling* automatically takes an abstract phenotype definition and translates it into a computable form. This naturally improves portability. Examples of this tooling include the *translators* developed by the PhEMA initiative, which are able to take a modelling language definition of a phenotype – such as definitions expressed in QDM, as produced by the PhAT, or in CQL – and transform them into executable formats (e.g pipelines [11]). In addition, the OHDSI tools provide ways to take their domain specific representation and translate it to SQL queries that execute against multiple database systems adopting the OMOP CDM. Although all definitions are SQL, the different dialects used by database vendors are akin to separate programming languages.

Given these benefits, a phenotype library should provide access to implementation tooling. In the simplest form, access should be provided to this tooling by hosting and indexing it in a library, in the same way that the definitions themselves are hosted and indexed. This tooling can then be downloaded, along with a definition, and executed locally in order to produce a computable form. More advanced integrations will provide the functionality offered by implementation tooling directly through the library, by running it as a service that can be accessed by users via the library in order to download the automatically generated computable form of a phenotype. This is the approach taken by the Phenoflow platform, which allows users to obtain computable copies of a phenotype definition directly, by running a microservice generation architecture.

The tooling indexed should be able to support implementations in a variety of different programming languages. While the programming language used might seem to be of little consequence, in practice, even with this presence of a translator, the researcher generating a computable form for a new use case is likely to still have to modify (localise) that computable phenotype for local use. Such modifications might include optimisations to the structure of the implementation to allow the computable form to operate in low-memory environments or to operate as a part of existing infrastructure (e.g. a clinical trial platform [36]). In this instance, having that definition in a language that the researcher is comfortable with editing is important. For example, the pipeline-based implementation originally produced by the PheMA translator only supports the KNIME format. As such, a researcher has to be comfortable with this format in order to make edits. To maximise portability, phenotype libraries should aim to support implementation tooling capable of producing executable definitions in multiple languages. An example of this is seen within the Phenoflow platform, where one can generate a workflow that utilises modules from a variety of languages, including Python and Javascript, with containerised environments supporting the straightforward execution of these units locally.

Support tooling that provides connectivity with multiple data standards

When a phenotype definition is translated by a piece of tooling into an executable form, it is typical for that definition to be tied to a given data source format, from which the resulting cohort is identified. In certain cases, that data format is always the same. For example, OMOP cohort definitions, when translated into a computable form (SQL), are always tailored for the OMOP CDM. While beneficial in the sense that this provides an automated translation process that works across sites, those sites must all adopt the OMOP CDM, which is not always ~~the case~~feasible. Instead, in reality, sites may use a variety of implementation formats, such as i2b2 and FHIR. For these reasons, phenotype libraries should index implementation tooling that not only supports multiple language implementations, but also supports the realisation of definitions for different data formats. Naturally, the more data source formats supported, the more portable the ~~definition~~ definitions stored within a library ~~is~~are. For example, the computable forms generated by PhEMA's translators can be tailored for a variety of local data formats, including FHIR and the OMOP CDM itself. Similarly, in the Phenoflow library, interacting with a data source is considered to be the first step in a phenotype's definition, and as such different *connectors* are available when generating the computable form of a definition. These connectors support a variety of different standards such as OMOP and i2b2, and plans are in place to support dataset specific standards, such as the standard used by UK Biobank (via tooling such as *Funpack* [37]).

The connector approach also provides a natural point at which to conduct any necessary (automatic) translation between the coding system adopted by a target data source, and the coding system expected by the implemented definition. For example, if the target datasource adopts Read codes, but the computable phenotype relies on sets of ICD codes, a connector might not only ingest data, but also perform code mappings accordingly.

Despite these benefits, the requirement to produce a new translator, or new connector, for each new data source format, is a natural drawback to each of these approaches. However, the advantages over manual translation are still clear.

Validation

Validating a phenotype definition involves confirming its accuracy. To do this, the cohort identified by a computable phenotype is typically compared to a *reference standard*, such as the cohort identified by manual chart review from the same patient population (a *gold standard*). The extent to which the two cohorts overlap determines the validity of the definition. While reference standards are a common means of phenotype validation, other techniques exist, and are listed in Table 2. Phenotype definitions that are shown to be accurate are considered to be of a higher quality. Therefore, phenotype libraries should facilitate the validation process.

Support a defined validation process

To support the validation of stored definitions, a phenotype library should have a clear and scalable process for the submission of existing validation information by a user, across a variety of the mechanisms shown in Table 2. This information can then be stored and presented alongside each definition. For example, the CALIBER library stores phenotypes as code sets (342, at the time of writing), with a view to providing a framework for the definition of consistent phenotypes, which can then be reused by care service providers for nation-wide EHR-based observational research [14]. Each definition in CALIBER appears alongside algorithmic information about the relationship between the code sets and key validation information. Specifically, the CALIBER library offers up to 6 different techniques, which are used to validate a single def-

Table 2. Phenotype validation mechanisms

Mechanism	Description	Example
Disease registries	Compare the phenotype cohort with those present in the registry.	Comparison of a diabetes phenotype cohort with those patients present in a diabetes registry (e.g. T1D exchange).
Chart review	Compare the phenotype cohort with the patients identified by manual chart review.	Comparison with a diabetes gold standard, produced by double manual chart review of patients.
Cross-EHR concordance	Compare percentage of cases identified by a phenotype across different sources, and identify any overlap.	Comparison of the percentage of patients identified by a diabetes phenotype in primary and secondary care EHRs, and the identification of any case overlap.
Risk factors	Compare the magnitude of the phenotype cohort with standard risk calculations.	Comparison with the output of a Cox hazards model.
Prognosis	Compare the magnitude of the phenotype cohort with external prognosis models.	Comparison with a survival analysis.
Genetic associations	Compare whether the presence of a patient in a phenotype cohort is consistent with their genetic profile.	A patient is more likely to be a valid member of a diabetes cohort if they have the HLA-DR3 gene.

inition. Similarly, the ~~proposed~~ OHDSI gold standard phenotype library is so-called because there ~~are plans to implement~~ is a well-defined process proposed for the submission of phenotypes based on different user roles. Specifically, the submission of a computable phenotype definition to the library ~~will~~ (which can occur using the APHRODITE ~~architecture and~~ framework) will require definitions to be submitted by those in the *author* role, vetted by *librarians*, validated by users who act as *validators* and used by standard *users* [38].

Automate multiple validation techniques

When new definitions are submitted without validation information to a library, it should seek to automatically validate these definitions by comparing them, or their outputs, against assets that are hosted alongside the definitions, such as gold standard datasets. For example, in [12], the authors present *electronic phenotyping validation*, a framework for the automated comparison of a definition with manual chart review results. In the absence of such assets, a portal might host tooling designed to derive these assets automatically. For example, *PheValuator* trains a linear model based upon cases and controls identified using some of the techniques already discussed, such as the presence (or absence) of a large number of clinical codes relating to a certain condition within a patient's record [39]. This model is used, in turn, to construct an evaluation cohort, which matches each individual in the cohort to a probability value indicating the likelihood of them having the condition of interest. This cohort can then act as a *silver* standard, against which phenotype definitions can be validated, in this case by using the matched probabilities to construct totals from which sensitivity, specificity and positive predictive value (PPV) are calculated.

There is also an argument for the automated combination of different validation approaches, to avoid the shortcomings of each individual approach. For example, using a disease registry approach alone as a gold standard for phenotypes related to that disease, is not scalable or feasible for patient cohorts focusing on multimorbidities and complex demographic criteria. Similarly, validating using clinical notes ~~reviews~~review, where phenotype patient matches are manually reviewed, are not sustainable for large LHS infrastructures. While the manual text extraction of phenotypes can be effective in smaller scenarios, it is heavily dependent on the human expert and the sample being analysed, and not well-suited to cross-site studies with differences in clinical and operational procedures and opinion between sites.

As such, phenotype libraries should offer novel hybrid approaches to validation that encompass structured data, free text, and ancillary sources for both structured and unstructured data.

Enable feedback

To facilitate any (informal) user-based validation of stored definitions, a phenotype library should support social interactions between the authors and researchers that use it, with a view to providing authors with feedback and allowing them to address this feedback accordingly. Social functionality is supported by the Phenotype Knowledge Base (PheKB), which currently hosts around 70 phenotype definitions [13]. For example, within the library, users are able to post comments or questions against different phenotypes. A researcher can also request collaboration on the development of phenotype definitions.

However, those users permitted to interact with a phenotype definition within a portal may be restricted. Within PheKB, only users with certain organisational affiliations (e.g. the ~~eMerge~~ eMERGE nerwork or the Phenome-Wide Association Studies (PheWAS) community [40]) are provided with access by default, with other users required to request an account prior to providing feedback on definitions. Other portals may restrict access to different countries or regions.

In many cases, these restrictions are necessary during the development of a phenotype. For example, APHRODITE's definition repositories are kept private while they are still under development. However, once developed, definitions can be accessed through the repository via any web browser or through an R ~~shiny~~ Shiny app. Based on practices such as these, phenotype libraries should limit the restrictions they place on those who can engage with the definitions in phenotype libraries, once developed. By eliciting comments on the validity of hosted definitions from a wider audience, one is likely to gain a greater understanding as to the quality of a definition.

Sharing and Warehousing

Once a phenotype definition is appropriately reproducible, portable and validated, it should then be accessible for use by others. While the traditional and default role of a phenotype library is to provide such access, this can be optimised, as discussed in the following sections.

Expose a standard API

To maximise accessibility, a phenotype library should facilitate user interactions via multiple interfaces. The definitions in a library are usually available via a single interface: a graphical front-end. While this provides a reasonable baseline for accessibility, it does not maximise it. For example, a user cannot instruct a piece of software to interact with the library, to include definitions directly within a piece of code, resulting in potential inconsistencies arising from manual entry. Similarly, existing software systems, such as decision-support systems, cannot autonomously access

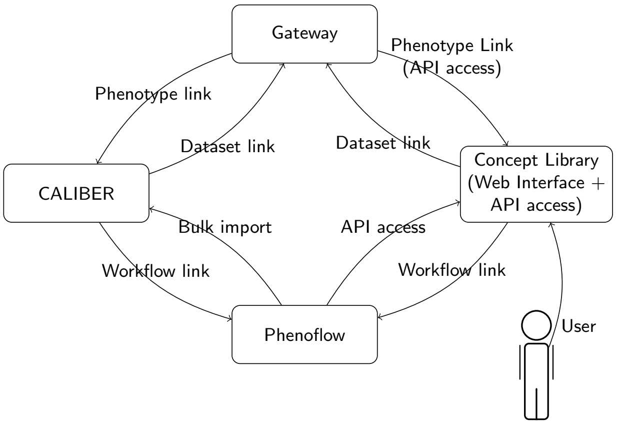

Figure 4. Overview of the services that constitute the HDR UK phenotype library

phenotypic information. Perhaps most importantly, a lack of programmatic accessibility means that one library cannot easily access the functionality of another in order to provide complimentary functionality.

To address these issue, phenotype libraries should offer API-level web services that (at a minimum) duplicate the functionality available in a user interface. In doing so, several considerations should be made. Firstly, the level of API access needs to be considered, including whether to provide access only to trusted *partners*, and thus provide suitable authentication mechanisms (e.g. OAuth), or whether to make the API publicly accessible. The selection of the type of API level access provided to the functionality of the web resource should be subject to the policy of the organisation developing the library. Secondly, the protocol used to facilitate communication with the API should be considered, such as Remote Procedure Call (RPC), Service Object Access Protocol (SOAP) and Representation State Transfer (REST). REST is a simple and widely adopted specification model [41], and is thus the technology that is likely to be most attractive when constructing a library API. Next, to support programmatic access and enable definitions to be differentiated automatically, a formal identification system should be established for each definition. The most straightforward way to this is to leverage the UUID attributed to each phenotype version.

The functionality of the API itself also needs to be considered. In [1], the authors propose that an API service should be used to construct phenotype definitions for the purpose of defining inclusion and exclusion criteria for clinical research trials. Building on this outline, we consider several additional API level use cases, including: searching phenotype definitions, extracting a specific phenotype definition, submitting a new phenotype definition, submitting a new use case for an existing phenotype definition or validating an existing definition and linking a phenotype definition with a data source, and vice-versa. Examples of specific functionality that an API level phenomics resource should support within each of these use cases is given in Table 3.

The benefits of API functionality are evident in the CALIBER, Phenoflow and Concept Library libraries, each of which communicate together to collectively form, along with a dataset Gateway, the HDR UK phenomics resource. As shown in Figure 4, each library operates as service, and collectively these services are able to deliver the functionality of a single library to a user. The services at the core of this library are the Concept Library and the CALIBER library, each of which store phenotype definitions. Using provided APIs, the Concept Library is able to import definitions from the CALIBER library, enabling phenotypes to be both formally stored and validated across both services, respectively. Similarly, the Phenoflow service – also capable of automatically importing and representing definitions using a workflow-based model, and generating a corresponding computable form for execution against a local dataset – is able to import definitions from both the Concept Library and CALIBER. Finally, the Gateway service provides access to a comprehensive collection of datasets, which are linked to by services such as CALIBER, when a given phenotype definition is present in one of the hosted datasets. Similarly, the Gateway links back to CALIBER when a phenotype is present in a dataset, in order to facilitate searches based upon these definitions.

Offer advanced search capabilities

The accessibility of existing phenotypes within a library relies on its search capabilities. Searches based on given name or identifier and version should enable simple use cases. For example, PheKB offers comprehensive search functionality, with users not only able to perform searches against the definitions themselves using given keywords, but also against supporting definition content, such as articles, implementations and datasets. Alternatively, the library has the option to list all phenotype definitions – including phenotype definitions under development, if the user is logged in – where a user can instead filter the definitions returned after the fact, based on properties such as the authoring institution.

While the search functionality offered by PheKB is helpful, more advanced search capabilities should be supported to facilitate both more complex cases and improved information retrieval. This includes searches based on specific codes, or groups of codes, or an approximate pattern matching, based on regular patterns or even text similarity. Synonyms (including abbreviations and acronyms) may also be used as a mechanism to improve search results over keyword searches. For example, a search for 'diabetes' would likely fail to find a phenotype that refers to 'T2DM' throughout, although 'T2DM' is a recognised abbreviation that can be semantically linked via the UMLS.

Even more advanced capabilities might include searches employing semantic similarity between a given set of concepts and the stored phenotypes supported by phenotype ontologies [42]. This could enable the discovery of semantically identical or closely related concepts within the library. Similarly, similarity metrics between phenotype definitions, facilitated by the adoption of a formal phenotype model, are likely assist in scalable searches across different repositories, whereby a partial match may indicate a usable cohort definition to investigate.

Include comprehensive metadata

The search and browse features described must be supported by appropriate metadata, which can be used to describe both the subject and format of phenotypes in ways that make them findable to users with specific research or clinical needs. Such definitions we might refer to as 'FAIR Phenotypes' [43]. To achieve this, each phenotype definition should include structured data that describes the subject (i.e., clinical condition) and intent (screening, etc.) of the definition, as well as the source, date, publisher, etc., similar to the tagging of resources in traditional libraries. Additionally, each component of the phenotype model (e.g., underlying data model, data elements, value sets, code lists, coding language) must be specified with an assigned code or value so that users can search on these features or have them displayed when browsing a phenotype library or repository. Examples of existing libraries that look to attribute appropriate metadata to stored definitions include CALIBER and PheKB (Figure 5).

In addition to supporting search, the use of metadata is important for a number of other reasons. Firstly, metadata can make clear characteristics of phenotypes related to their accessibility, interoperability and re-use. To this end, as part of the Mobilizing Computable Biomedical Knowledge (MCBK) initiative, Alper and Flynn et al. have proposed 12 categories of metadata that are re-

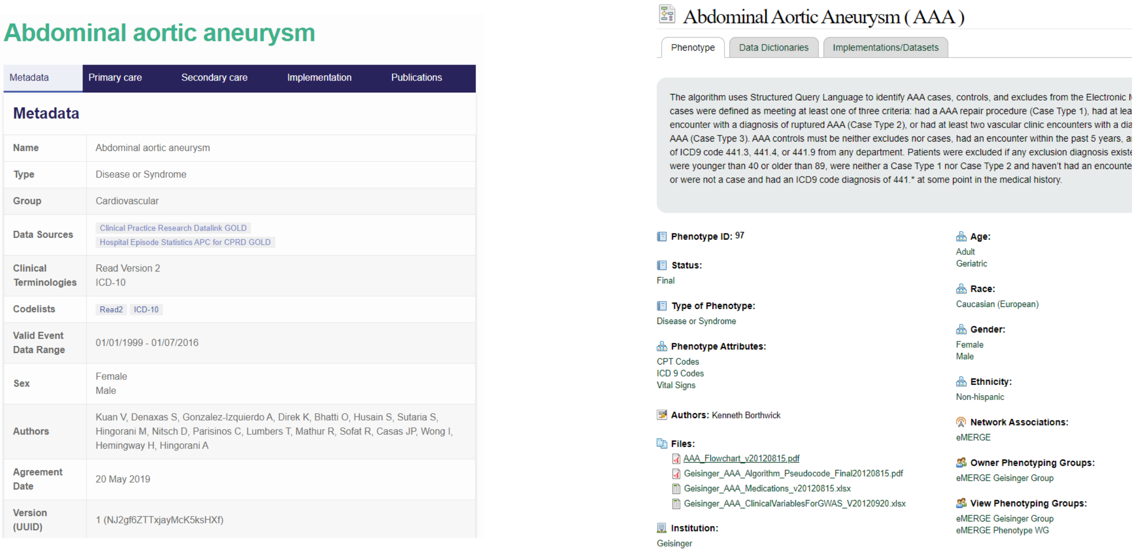

Figure 5. Metadata structure adopted by CALIBER (left) and PheKB (right).

quired to fully represent knowledge objects, including phenotypes, for FAIR principled criteria [44]. In addition, metadata fields that describe the versioning aspects of a definition can be populated to further formalise the provenance of the phenotypes in a collection. Next, as the intent, development, and validation of phenotypes are essential for potential implementers to understand in order to trust the quality and appropriateness of a phenotype for a new purpose, representing aspects of the pheontype development and validation process formally is critical. To do this, the Trust and Policy Work Group of the Patient-Centered Clinical Decision Support Learning Network defined an extensive set of metadata for trust [45]. Finally, metadata can be used to formally represent many aspects of the implementation and tooling described, enabling potential implementers to search on these features, such as language, and possibly support automated translations.

While more and robust metadata are beneficial from a library perspective, populating these metadata accurately and consistently ~~require~~ requires resources, and the extent and detail of metadata will depend upon a balance to adequately meet the needs at the expense that the library sponsor will bear. One potential solution to this issue is to automatically generate metadata, which is the approach taken in data management platforms [46]. Overall, time will show how the community of phenotype users can develop consensus on a minimum set of metadata, library or indexing best practices to complement and formalise the desiderata described here, and also build a compelling value case for their use to support high quality phenotyping across countries.

Conclusions

While making significant advances, computable phenotyping is still at an early stage where methods and repositories are emerging to meet the needs of a range of medical research domains, with little methodological consensus. As tooling gradually matures beyond the realm of early adopters to become usable for a broad spectrum of researchers and implementers, the focus needs to move away from one-size-fits-all 'perfect' phenotype definitions to acknowledging the diversity of phenotype application areas, the resultant explosion in the numbers and variations of phenotypes to be stored ~~, and~~ – in particular the arrival of advanced probabilistic and NLP-based phenotypes to sit alongside traditional rule-based definitions – and the challenges of deploying them in the real world, especially in the presence of high-throughput requirements. Portability and reproducibility are essential in addressing this scaling-up, with techniques needed to move phenotype definitions between both data sources and different health settings.

Phenotype libraries offer a natural meeting point of these multiple use-cases and domains to support high-quality phenotype definitions. In terms of designing phenotype libraries as technical entities that enable the storage and retrieval of definitions, there is a clear need to track the evolution of phenotype definitions as they are authored, support advanced search techniques that enable these definitions to be located by others, and establish a collaborative process through which the validity of definitions can be critiqued. All of this functionality should be accessible within a library via multiple channels, in particular comprehensive, standards-based API functionality to ensure interoperability. Authoring and storing phenotype definitions according to a standard model is another aspect through which phenotype libraries can contribute to definition reproducibility. The model adopted by a phenotype library should exist at the correct level of abstraction, prioritising modelling languages over executable programming languages, and offset this, in terms of implementation, by incorporating key implementation information, and improving clarity through multi-dimensional descriptions. Finally, a phenotype library should encourage the use of phenotype definitions in new use cases by supporting the validation process, both automatically, and through the definition of a structured validation process.

The impact of supporting the development and implementation of high-quality phenotype definitions is significant, particularly as these definitions provide efficient access to accurate cohort data by overcoming many of the complexities associated with patient datasets. Cohort data not only supports research studies (e.g. the identification of predictors for a certain condition), but also the provision of decision support (e.g. access to the medical histories of one or more individuals) and clinical trials (e.g. the establishment of trial cohorts). The use of computable phenotypes to determine cohorts from complex datasets for these purposes can be complemented by using traditional big data techniques to manage scale; by an increased focus on multi-morbidities – the complex interactions of diseases in patients – which are a crucial factor in personalised decision support systems; and by *N-of-1* clinical trial design.

Overall, running through these desiderata is the awareness that cross-domain sharing of phenotype definitions can only occur through curated libraries that evolve in a controlled manner. Such libraries have to be 1) clinically and scientifically valid; 2) techni-

cally realisable; and 3) usable by researchers in different domains. Through the usage of our desiderata, we believe the current and future phenotype libraries will deliver on these three fronts.

Declarations

List of abbreviations

Automated PHenotype Routine for Observational Definition, Identification, Training and Evaluation (APHRODITE); Clinical Quality Language (CQL); Electronic Health Record (EHR); Mobilizing Computable Biomedical Knowledge (MCBK); Observational Health Data Sciences and Informatics (OHDSI); PhEMA Authoring Tool (PhAT); Phenotype Knowledge Base (PheKB); Phenotyping pipeline (PheP); Phenome-Wide Association Studies (PheWAS); Phenotype Execution and Modelling Architecture (PhEMA); Quality Data Model (QDM).

Consent for publication

Not applicable.

Competing Interests

The author(s) declare that they have no competing interests.

Funding

This work was supported by Health Data Research UK, which receives its funding from Health Data Research UK Ltd (NIWA1; GVG and AK: HDRUK/CFC/01) funded by the UK Medical Research Council (MRC), Engineering and Physical Sciences Research Council, Economic and Social Research Council, Department of Health and Social Care (England), Chief Scientist Office of the Scottish Government Health and Social Care Directorates, Health and Social Care Research and Development Division (Welsh Government), Public Health Agency (Northern Ireland), British Heart Foundation, and the Wellcome Trust. In addtion, SD acknowledges that this study is part of the BigData@Heart programme that has received funding from the Innovative Medicines Initiative 2 Joint Undertaking (116074), which receives support from the European Union's Horizon 2020 research and innovation programme (H2020) and the European Federation of Pharmaceutical Industries and Associations (EFPIA). MC and VC are supported by the National Institute for Health Research (NIHR) Biomedical Research Centre based at Guy's and St Thomas' National Health Service Foundation Trust and King's College London, and the Public Health and Multimorbidity Theme of the National Institute for Health Research's Applied Research Collaboration (ARC) South London. GVG and AK also acknowledge support from the NIHR Birmingham Experimental Cancer Medicine Centre (ECMC), the NIHR Birmingham Surgical Reconstruction Microbiology Research Centre (SRMRC) and the NIHR Birmingham Biomedical Research Centre, as well as Nanocommons H2020 (731032) and an MRC fellowship grant (MR/S003991/1). HP acknowledges support from European Molecular Biology Laboratory (EMBL) core funds. LVR and JAP acknowledge support from the National Institute of General Medical Sciences (R01GM105688) and the National Human Genome Research Institute (U01HG011169).

The opinions in this paper are those of the authors and do not necessarily reflect the opinions of the funders.

Authors' Contributions

MC: conceptualisation, methodology, investigation, writing - original draft; SM: conceptualisation, methodology, investigation, writing - original draft; LVR: methodology, writing - original draft; AK: investigation, writing - original draft; GVG: investigation, writing - original draft; CG: investigation, writing - review & editing; DT: investigation, writing - review & editing; JAP: methodology, writing - review & editing; HP: writing - review & editing; RR: investigation, writing - original draft, writing - review & editing; EJ: funding acquisition, writing - review & editing; SD: methodology, funding acquisition, writing - review & editing; VC: funding acquisition, writing - original draft, writing - review & editing;

Acknowledgements

The authors wish to acknowledge the involvement of Dr Susheel Varma (HDR UK) and Dr Elliot Fairweather (King's College London) in the development of this work.

References

1. Richesson R, Smerek M, Cameron CB. A Framework to Support the Sharing and Re-Use of Computable Phenotype Definitions Across Health Care Delivery and Clinical Research Applications. eGEMs (Generating Evidence & Methods to improve patient outcomes) 2016;4(3):10–24.
4. ~~Cho K, Introduction to the VA Phenomics Library (VAPheLib);. Available at: , Accessed on: 2021-02-12.~~
3. Weaver J, Potvien A, Swerdel J, Voss EA, Hester L, Shoaibi A, et al. Best Practices for Creating the Standardized Content of an Entry in the OHDSI Phenotype Library. In: 5th OHDSI Annual Symposium; 2019. p. 46.
4. Cho K, Introduction to the VA Phenomics Library (VAPheLib);. Available at: "https://www.hsrd.research.va.gov/for_researchers/cyber_seminars/archives/3814-notes.pdf", Accessed on: 2021-02-12.
5. Meineke F, Stäubert S, Löbe M, Uciteli A, Löffler M. Design and Concept of the SMITH Phenotyping Pipeline. Studies in health technology and informatics 2019;267:164–172.
6. Clarivate Analytics, Web of science;. Available at: http://www.webofknowledge.com, Accessed 2021-02-15.
7. Pacheco JA, Rasmussen LV, Kiefer RC, Campion TR, Speltz P, Carroll RJ, et al. A case study evaluating the portability of an executable computable phenotype algorithm across multiple institutions and electronic health record environments. Journal of the American Medical Informatics Association 2018;25(11):1540–1546.
8. Brandt PS, Kiefer RC, Pacheco JA, Adekkanattu P, Sholle ET, Ahmad FS, et al. Toward cross-platform electronic health record-driven phenotyping using Clinical Quality Language. Learning Health Systems 2020;4(4):9–17.
9. Hripcsak G, Shang N, Peissig PL, Rasmussen LV, Liu C, Benoit B, et al. Facilitating phenotype transfer using a common data model. Journal of Biomedical Informatics 2019;96:120–127.
17. Banda JM, Halpern Y, Sontag D, Shah NH. Electronic phenotyping with APHRODITE and the Observational Health Sciences and Informatics (OHDSI) data network. In: Proceedings of AMIA Joint Summits on Translational Science; 2017. p. 48–57.
11. Mo H, Pacheco JA, Rasmussen LV, Speltz P, Pathak J, Denny JC, et al. A Prototype for Executable and Portable Electronic Clinical Quality Measures Using the KNIME Analytics Platform. In: Proceedings of AMIA Joint Summits on Translational Science, vol. 2015; 2015. p. 127–31.
12. Kukhareva P, Staes C, Noonan KW, Mueller HL, Warner P,

Shields DE, et al. Single-reviewer electronic phenotyping validation in operational settings: Comparison of strategies and recommendations. Journal of Biomedical Informatics 2017;66(C):1–10.
13. Kirby JC, Speltz P, Rasmussen LV, Basford M, Gottesman O, Peissig PL, et al. PheKB: a catalog and workflow for creating electronic phenotype algorithms for transportability. Journal of the American Medical Informatics Association 2016;23(6):1046–1052.
14. Denaxas S, Gonzalez-Izquierdo A, Direk K, Fitzpatrick NK, Fatemifar G, Banerjee A, et al. UK phenomics platform for developing and validating electronic health record phenotypes: CALIBER. Journal of the American Medical Informatics Association 2019;26(12):1545–1559.
15. Chapman M, Rasmussen L, Pacheco J, Curcin V. Phenoflow: A Microservice Architecture for Portable Workflow-based Phenotype Definitions. In: Proceedings of AMIA Joint Summits on Translational Science; 2021. p. 142–151.
16. SAIL Databank, The Concept Library;. Available at: https://conceptlibrary.demo.saildatabank.com/home/, Accessed on: 2021-02-11.
17. Banda JM, Halpern Y, Sontag D, Shah NH. Electronic phenotyping with APHRODITE and the Observational Health Sciences and Informatics (OHDSI) data network. In: Proceedings of AMIA Joint Summits on Translational Science; 2017. p. 48–57.
Clarivate Analytics, Web of science;. Available at: , Accessed 2021-02-15.
18. Boxwala AA, Rocha BH, Maviglia S, Kashyap V, Meltzer S, Kim J, et al. A multi-layered framework for disseminating knowledge for computer-based decision support. Journal of the American Medical Informatics Association 2011;18(Supplement_1):132–139.
19. Curcin V. Why does human phenomics matter today? Learning Health Systems 2020;4(4):1–3.
20. Sá-Sousa A, Jacinto T, Azevedo LF, Morais-Almeida M, Robalo-Cordeiro C, Bugalho-Almeida A, et al. Operational definitions of asthma in recent epidemiological studies are inconsistent. Clinical and Translational Allergy 2014;4(1):24.
21. Mo H, Thompson WK, Rasmussen LV, Pacheco JA, Jiang G, Kiefer R, et al. Desiderata for computable representations of electronic health records-driven phenotype algorithms. Journal of the American Medical Informatics Association 2015;22(6):1220–1230.
22. Papez V, Denaxas S, Hemingway H. Evaluating OpenEHR for Storing Computable Representations of Electronic Health Record Phenotyping Algorithms. In: Proceedings - IEEE Symposium on Computer-Based Medical Systems; 2017. p. 509–514.
23. Jiang G, Prud'Hommeaux E, Xiao G, Solbrig HR. Developing A Semantic Web-based Framework for Executing the Clinical Quality Language Using FHIR. In: CEUR Workshop Proceedings; 2017. p. 126–130.
24. Agarwal V, Podchiyska T, Banda JM, Goel V, Leung TI, Minty EP, et al. Learning statistical models of phenotypes using noisy labeled training data. Journal of the American Medical Informatics Association 2016;23(6):1166–1173.
25. Yu S, Ma Y, Gronsbell J, Cai T, Ananthakrishnan AN, Gainer VS, et al. Enabling phenotypic big data with PheNorm. Journal of the American Medical Informatics Association 2018;25(1):54–60.
26. Banda JM, Seneviratne M, Hernandez-Boussard T, Shah NH. Advances in electronic phenotyping: from rule-based definitions to machine learning models. Annual review of biomedical data science 2018;1:53–68.
27. Zheng NS, Feng Q, Eric Kerchberger V, Zhao J, Edwards TL, Cox NJ, et al. PheMap: A multi-resource knowledge base for high-throughput phenotyping within electronic health records. Journal of the American Medical Informatics Association 2020;27(11):1675–1687.
28. Cimino JJ. Desiderata for controlled medical vocabularies in the twenty-first century. Methods of Information in Medicine 1998;37(04/05):394–403.
29. Banda JM, Williams A, Kashyap M, Seneviratne MG, Potvien A, Duke J, et al. FAIR Phenotyping with APHRODITE. In: 5th OHDSI Annual Symposium; 2019. p. 45.
30. Curcin V, Fairweather E, Danger R, Corrigan D. Templates as a method for implementing data provenance in decision support systems. Journal of Biomedical Informatics 2017;65:1–21.
31. Moreau L, Missier P, Belhajjame K, B'Far R, Cheney J, Coppens S, et al. PROV-DM: The PROV Data Model. World Wide Web Consortium; 2013.
32. Fairweather E, Chapman M, Curcin V. A delayed instantiation approach to template-driven provenance for electronic health record phenotyping. In: Proceedings of the 9th International Provenance and Annotations Workshop, IPAW 2021 (In press); 2021. .
33. Castro VM, Minnier J, Murphy SN, Kohane I, Churchill SE, Gainer V, et al. Validation of electronic health record phenotyping of bipolar disorder cases and controls. American Journal of Psychiatry 2015;172(4):363–372.
34. FinnGen, Risteys: Explore FinnGen data at the phenotype level;. Available at: https://risteys.finngen.fi/, Accessed 2021-03-05.
35. Xu Z, Wang F, Adekkanattu P, Bose B, Vekaria V, Brandt P, et al. Subphenotyping depression using machine learning and electronic health records. Learning Health Systems 2020;4(4):40–49.
36. Chapman M, Domínguez J, Fairweather E, Delaney BC, Curcin V. Using Computable Phenotypes in Point-of-Care Clinical Trial Recruitment. In: Digital Personalized Health and Medicine - Proceedings of MIE 2021 (In press); 2021. .
37. McCarthy P, funpack. Zenodo; 2021. 10.5281/zenodo.4646309.
38. Knoll C, Banda J, Rao G, Chen R, Swerdel J. OHDSI Gold Standard Phenotype Library. Observational Health Data Sciences and Informatics; 2019.
39. Swerdel JN, Hripcsak G, Ryan PB. PheValuator: Development and evaluation of a phenotype algorithm evaluator. Journal of Biomedical Informatics 2019;97.
40. Denny JC, Ritchie MD, Basford MA, Pulley JM, Bastarache L, Brown-Gentry K, et al. PheWAS: demonstrating the feasibility of a phenome-wide scan to discover gene–disease associations. Bioinformatics 2010;26(9):1205–1210.
41. Fielding RT. Architectural styles and the design of network-based software architectures. PhD thesis, University of California, Irvine; 2000.
42. Gkoutos GV, Schofield PN, Hoehndorf R. The anatomy of phenotype ontologies: principles, properties and applications. Briefings in bioinformatics 2018;19(5):1008–1021.
43. Wilkinson MD, Dumontier M, Aalbersberg IJ, Appleton G, Axton M, Baak A, et al. The FAIR Guiding Principles for scientific data management and stewardship. Scientific Data 2016;3(1):1–9.
44. Alper B, Flynn A, Bray B. Categorizing Metadata to Help Mobilize Computable Biomedical Knowledge. Learning Health Systems (Under Review) 2021;.
45. Richardson JE, Middleton B, Platt JE, Blumenfeld BH. Building and maintaining trust in clinical decision support: Recommendations from the Patient-Centered CDS Learning Network. Learning Health Systems 2020;4(2):7–14.
46. Nind T, Galloway J, McAllister G, Scobbie D, Bonney W, Hall C, et al. The research data management platform (RDMP): A novel, process driven, open-source tool for the management of longitudinal cohorts of clinical data. GigaScience 2018;7(7):1–12.

Table 3. Suggested library API functions. All requests made in, and responses returned in, YAML+Markdown/JSON/XML formats.

Function		User Access Level	Description
Search	Simple Search	Public	A free text search, examining the entire contents of the portal and returning a list of phenotypes that match the search criteria.
	Advanced Search	Public	A free text search, examining specified sections of the portal (e.g., main content, just metadata, etc.) and returning a list of phenotypes that match the search criteria.
Phenotype extraction	Extracting specific phenotype(s)	Public	Given a phenotype ID supplied by a user (or generated by the platform), the API returns the phenotype definition.
	Extracting all phenotypes	Public	Return a full list of phenotypes.
Adding new phenotype(s)		Authorised users	Only authorised users should be allowed to either submit a single or group of phenotype definitions.
Updating a phenotype definition	Updating the contents of a specific phenotype	Authorised users	Each aspect of a phenotype definition – including constituent codelists, links to datasets where that phenotype appears, and other metadata – can be updated by passing a phenotype ID and the names of the fields to update and their new values. Each update should mark a version number to keep record of any updates over time.
	Updating a complete phenotype with multiple features	Authorised users	Update a phenotype contents by passing a phenotype ID and submitting an updated phenotype definition file to replace the previous version for public view.
	Submission of a new validation case study for an existing phenotype	Authorised users	Adding a new use case to validate an existing phenotype (identified by a phenotype ID) by passing a file.
Deletion of a phenotype	Removing a phenotype from public view (soft delete)	Private to portal administrators	An administrator of the portal can hide a phenotype definition by providing a phenotype ID.
	Removing a phenotype from the library (hard delete).	Private to portal administrators	An administrator of the portal can delete a phenotype definition entirely by providing a phenotype ID.

Manuscript Click here to access/download;Manuscript;main.tex

GigaScience, 0000, 1–14

doi: xx.xxxx/xxxx
Manuscript in Preparation
Review

REVIEW

Desiderata for the development of next-generation electronic health record phenotype libraries

Martin Chapman [0000-0002-5242-9701][1,*], Shahzad Mumtaz [0000-0003-2606-2405][2], Luke V. Rasmussen [0000-0002-4497-8049][3], Andreas Karwath [0000-0002-6942-3760][4], Georgios V. Gkoutos [0000-0002-2061-091X][4], Chuang Gao [0000-0002-6456-0301][2], Dan Thayer [0000-0003-1847-4362][5], Jennifer A. Pacheco [0000-0001-8021-5818][3], Helen Parkinson [0000-0003-3035-4195][6], Rachel L. Richesson [0000-0003-0279-7036][7], Emily Jefferson [0000-0003-2992-7582][2], Spiros Denaxas [0000-0001-9612-7791][8] and Vasa Curcin [0000-0002-8308-2886][1]

[1]King's College London, London, UK and [2]Health Informatics Centre (HIC), University of Dundee, Dundee, UK and [3]Northwestern University Feinberg School of Medicine, Chicago, Illinois, USA and [4]University of Birmingham, Birmingham, UK and [5]SAIL Databank, Swansea, UK and [6]European Molecular Biology Laboratory - European Bioinformatics Institute (EMBL-EBI), Hinxton, UK and [7]University of Michigan Medical School, Michigan, USA and [8]University College London, London, UK

[*] Corresponding Author. Email: martin.chapman@kcl.ac.uk

Abstract

Background High-quality phenotype definitions are desirable to enable the extraction of patient cohorts from large electronic health record (EHR) repositories, and are characterised by properties such as portability, reproducibility and validity. Phenotype libraries, where definitions are stored, have the potential to contribute significantly to the quality of the definitions they host. In this work, we present a set of desiderata for the design of a next-generation phenotype library that is able to ensure the quality of hosted definitions by combining the functionality currently offered by disparate tooling. **Methods** A group of researchers examined work to date on phenotype models, implementation and validation, as well as contemporary phenotype libraries developed as a part of their own phenomics communities. Existing phenotype frameworks were also examined. This work was translated and refined by all the authors into a set of best practices. **Results** We present 14 library desiderata that promote high-quality phenotype definitions, in the areas of modelling, logging, validation and sharing and warehousing. **Conclusions** There are a number of choices to be made when constructing phenotype libraries. Our considerations distil the best practices in the field and include pointers towards their further development to support portable, reproducible, and clinically valid phenotype design. The provision of high-quality phenotype definitions enables EHR data to be more effectively used in medical domains.

Key words: Electronic health records; EHR-based phenotyping; computable phenotype; phenotype library

Compiled on: July 15, 2021.
Draft manuscript prepared by the author.

Key Points

- Portable, reproducible and clinically valid phenotype definitions have the potential to unlock health data repositories for wider and more effective use.
- To ensure definitions are of high quality, associated tools should be supported directly through the libraries where phenotype definitions are hosted.
- 14 desiderata are presented to guide the development of future phenotype libraries, and to ensure phenotype definitions are of a sufficient quality to enable the effective use of medical data in research and in healthcare provision.

Introduction

As a result of digitisation of health systems world-wide, electronic health record (EHR) data repositories have emerged as the main source of data for medical cohort research studies. To extract these cohorts, there is an increasing reliance on EHR-based *phenotype* definitions (also referred to as phenotyping algorithms), which identify individuals that exhibit certain phenotypic traits, such as the same diseases, characteristics, or set of co-morbidities. These definitions can be represented in many forms, including narrative descriptions, pseudo-code, or, in some cases, may already be directly executable. Conceptually, they may vary from simple code lists, via rule-based algorithms to more involved machine learning (ML) tasks and high-throughput approaches using natural language processing (NLP).

While traditional big data techniques can successfully address the scale of the EHR data available, the effectiveness of phenotype definitions is impacted by a range of other syntactic and semantic issues, including variations in the way data is structured and the coding systems used.

To overcome these issue and enable effective cohort extraction, a phenotype definition must exhibit certain properties. It must be *reproducible* allowing for accurate (re)implementation, irrespective of the idiosyncrasies of the dataset against which the definition was originally developed; *portable*, allowing for straightforward implementation, irrespective of the structure of the target dataset; and *valid*, effectively capturing the disease or condition modelled. A definition that exhibits all of these properties we refer to as *high quality*.

To ensure high-quality phenotype definitions, support should be provided to the authoring, implementation, validation and dissemination processes of a phenotype's *lifecycle*. While such support is currently available, it is often sporadic and inconsistent as it is delivered via a wide range of different tools. Instead, building on the work of Richesson et. al [1], we propose that the functionality provided by these tools should instead be provided centrally, through the phenotype *libraries* where definitions are hosted. For example, libraries should enable phenotypes to be developed according to some set of standard models, and track the evolution of definitions under these models, so as to ensure hosted definitions are clearer to understand and thus have the potential to be more reproducible. Moreover, libraries should assist in the derivation of directly computable phenotype definitions, through the provision of implementation tooling, to improve portability by enabling the execution of phenotypes in local use cases. Similarly, libraries should directly validate the definitions they host, through, for example, automated comparisons with gold standards.

To this end, in this work we contribute a number of desiderata for the development of phenotype libraries, which not only ensure that definitions are accessible, but also maximise the quality of the phenotypes they contain by supporting all parts of the definition lifecycle. These desiderata are based on both the lessons learned during the development of contemporary libraries within the authors' own phenomics communities, as well as a review of the functionality currently offered by phenotype tooling, which represent practices that have lead to the development of high-quality phenotype definitions. By providing access to high-quality definitions, phenotype libraries enable both efficient and accurate use of EHR data for activities such as medical research, decision support and clinical trial recruitment.

Background

Human phenomics is the study of human phenotypes, and includes the science and practice of defining observable medical phenomena that indicate phenotypes to advance research and personalised care. The concept of a phenotype originated as a complement to the genotype, and a phenome was defined as a complete set of an individual's inheritable characteristics. Rather than describing someone's genetic information, a phenome captures all the observable properties (phenotypes) that result from the interaction of their genetic make-up and environmental factors, including their demographic information, such as height or eye color, and medical histories.

With the emergence of large-scale EHR data repositories, the term phenotype has evolved to denote traits shared by groups of patients, such as a disease or condition that a cohort, or set of individuals, has. This may also include other complex combinations of traits, exposures, or outcomes, including co-morbidities, polypharmacy, and demographic data. Defining these phenotypes, and validating them to ensure their accuracy and generalisability, is the process known as *phenotyping*, with *EHR-based phenotyping* relying primarily on data in the EHR. *Computational phenotyping* (also known as *deep phenotyping*) uses either supervised machine learning techniques to discover new members of a priorly defined cohort, or unsupervised techniques to discover entirely new phenotypes and investigate their properties.

EHR data repositories bring with them a very specific set of data challenges in terms of managing syntactic and semantic complexity, which act as a barrier to studies that need to utilise patient information from across multiple data sources and for the needs of different studies. For example, by the nature of healthcare delivery and how EHRs are used to document, a patient who has been diagnosed with diabetes mellitus may be represented slightly differently in two EHR systems, and will almost certainly be represented differently in EHRs for different countries.

Phenotype *libraries* – where definitions can be uploaded, stored, indexed, retrieved, and downloaded by users – provide a logical place in which to ensure that definitions are of a suitable quality to overcome many of the issues associated with extracting cohorts from complex EHR datasets. This is accentuated by the

fact that the development of phenotype libraries is a rapidly growing area. Of particular note is the Observational Health Data Sciences and Informatics (OHDSI) Gold Standard Phenotype Library, which aims to support OHDSI community members in finding, evaluating and utilising cohort definitions that are validated by the research community. An initial version of the library is currently available, alongside a wider set of requirements to guide its future development [2]. Other libraries planned for development include the VAPheLib [3], which aims to collect, store and make available 1000 curated phenotype definitions for the clinical operations research community by the end of 2021. Phenotype libraries are also being developed as a part of wider phenotype frameworks. Alongside Richesson's reusable phenotype definition framework sit initiatives such as the *phenotyping pipeline* (PheP), which aims to extract, structure and normalise phenotypes from EHR data collected across participating sites [4].

Methods

To determine the functionality that should be provided by a next-generation phenotype library, a team of international researchers – comprising Health Data Research UK (HDR UK) Phenomics theme members and US researchers from the Mobilizing Computable Biomedical Knowledge (MCBK) and Phenotype Execution and Modelling Architecture (PhEMA) communities – first examined a range of tools supporting different parts of the definition lifecycle, which were developed within their respective phenomics communities. This was enriched with a wider review of the literature via Web of Science (WoS) [5] and the grey literature via Google to identify third-party projects that have developed phenotype tooling, or are planning its development, and future trends. Our decision to include the grey literature was informed by our a priori knowledge of tools under development that have not yet published peer-review articles. The tools reviewed included those that support authoring (e.g., modelling using the Quality Data Model (QDM) logic [6], the Clinical Quality Language (CQL) [7], and use of the Observational Medical Outcomes Partnership (OMOP) Common Data Model (CDM) [8] and associated tooling such as OHDSI's Automated PHenotype Routine for Observational Definition, Identification, Training and Evaluation (APHRODITE) [9]), implementation (e.g., definition translators [10]) and validation (e.g., electronic phenotyping validation [11]). Common functionality provided by the tools identified – representing opportunities for new phenotype library functionality – was extracted and summarised.

In addition, the authors examined existing libraries from within their own communities – including the Phenotype Knowledge Base (PheKB) [12], CALIBER [13], Phenoflow [14] and the Concept Library [15] – to identify instances of functionality currently supporting the phenotype definition lifecycle. Common functionality provided by these libraries – which has been shown to result in reproducible, portable and valid phenotype definitions, and thus represent best practice – was also extracted and summarised.

Both of these summaries were translated to a draft set of desiderata via discussion amongst a subset of the authors [MC, SM, EJ, SD, VC]. All authors participated in an asynchronous iterative review process to critique, consolidate, refine, and define the final set of desiderata. The desiderata were further classified into logical categories.

Desiderata

In total, the authors arrived at a finalised collection of 14 desiderata, which are organised across the following sections into five categories: modelling, logging, implementation, validation and sharing and warehousing. Figure 1 shows how the desiderata presented promote the design of a phenotype library that supports all parts of the phenotype definition lifecycle.

Modelling

Phenotype models govern the structure and syntax of phenotype definitions. For example, phenotype definitions are traditionally rule-based, meaning that they are comprised of individual logical statements that each evaluate to a boolean value, typically by relating *data elements* (with associated *values*) – such as the presence of a particular set of ICD-10 codes or a particular lab result – to each other. The set of operators available to an author when connecting data elements (e.g. logical connectives such as conjunction and disjunction) would be established within a phenotype definition model. A model may dictate that a phenotype be represented in an unstructured, semi-structured, structured, or executable manner [16]. A summary of different phenotype definition formats, governed by phenotype models, is given in Table 1.

Implementing a phenotype definition involves translating the abstract definition (if unstructured or semi-structured) into an executable form that can be directly run against a patient dataset in order to derive the cohort exhibiting the defined phenotype. Typically this requires the logic of the definition to be realised in a programming language, such as translating abstract conditional clauses into a set of tangible Python conditional statements. We refer to these implementations as *computable phenotypes*. For a definition to be reproducible, it must be realised in a formal structure that can be accurately interpreted and implemented. Given the potential for human error in translating from an unstructured narrative to something computable, formal phenotype models provide such a structure.

Phenotype models are also key in ensuring semantic interoperability between definitions. That is, while the development of phenotype definitions can involve deriving a curated, canonical set of phenotype definitions containing 'definitive' versions for each disease of condition being modelled for a particular domain (e.g. a national stroke body may want to maintain their set of stroke phenotyping algorithms), more often than not, it is perfectly valid to have overlapping phenotype definitions for different uses. For example, an eligibility criteria for a clinical trial may differ from a rule that triggers a decision support tool in an EHR system, and both would differ from a definition used in a population health study, even if all three nominally refer to same disease [17]. Internationally, definitions for the same disease may also differ [18]. While this overlap is permissible, different definitions for the same condition must still be compatible, enabling, for example, their relative functionality to be compared. The adoption of a phenotype model enables such compatibility.

Given these benefits, a phenotype library should adopt a formal phenotype model to control the structure of hosted definitions. To ensure the use of such a model, a library can offer a graphical authoring environment – in the same way that tools such as the Phenotype Execution and Modelling architecture (PhEMA) Authoring Tool (PhAT) do [6] – through which new definitions can be authored. Similarly, existing definitions can be automatically checked for their adherence to the chosen model when uploaded.

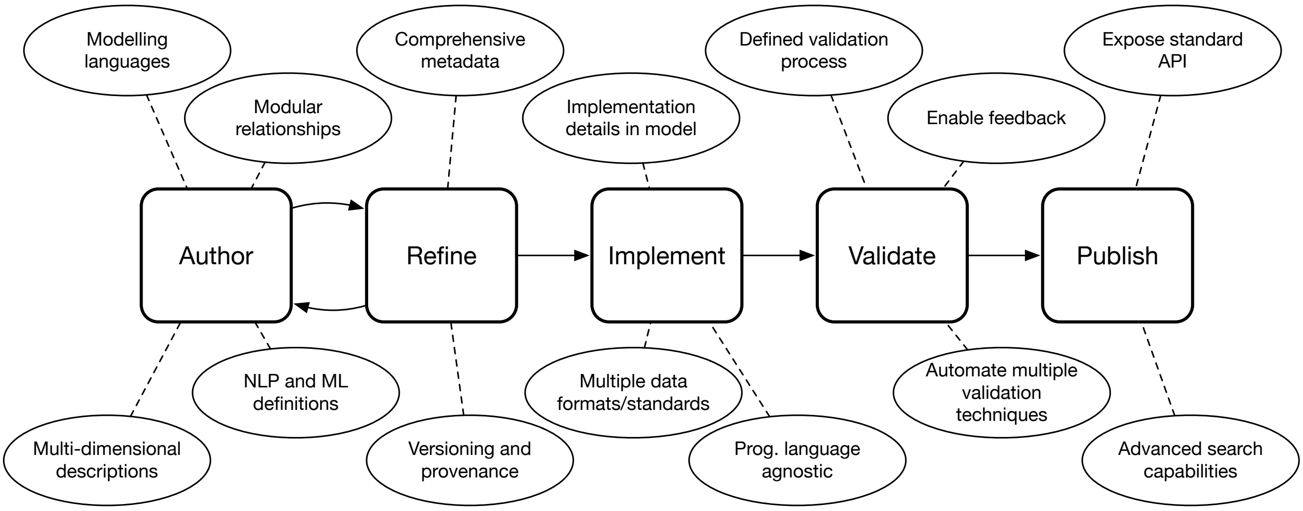

Figure 1. The stages of the phenotype definition lifecycle supported by a next-generation phenotype library.

Table 1. Phenotype definition formats

Format	Description	Example	Category
Code list	A set of codes that must exist in a patient's health record in order to include them within a phenotype cohort	COVID-19 ICD-10 code *U07.1*	Rule-based
Simple data elements	Formalising the relationship between code-based *data elements* using *logical connectives*	COVID-19 ICD-10 code *U07.1* AND ICD-11 code *RA01.0*	Rule-based
Complex data elements	Formalising the relationship between complex data elements, such as those derived via NLP.	Patient's blood pressure reading > 140 OR patient notes contain 'high BP'	Rule-based
Temporal	Prefix rules with temporal qualifiers	Albumin levels increased by 25% over 6 hours, high blood pressure reading has to occur during hospitalisation.	Rule-based
Trained classifier	Use rule-based definitions as the basis for constructing a classifier for future (or additional) cohorts	A k-fold cross validated classifier capable of identifying COVID-19 patients	Probabilistic

Desiderata relating to the adoption of a phenotype model by a library are listed in the following sections. We view these desiderata as complementary to the well-established desiderata for phenotype definition model development put forward by Mo et al. [19].

Support modelling languages

The phenotype definition model adopted by a library should be supported by a (non-executable) high-level modelling language that dictates the syntax available to an author when defining the logic of a phenotype. A computable form of the definition can then be realised for execution in a local use case. When selecting or developing a definition model, the temptation may be to select a lower-level, executable programming language, in an attempt to expedite local implementation. For example, one could argue that a language such as Python is sufficient for simultaneously defining phenotypes and realising them computationally. However, we would argue that using such a language as a means to express the logic of a definition ties the definition to general purpose, low-level language constructs, reducing clarity, and thus reproducibility. This conclusion is supported by work such as [20], which found openEHR an overly restrictive standard when attempting to express phenotype definitions in a form that can be directly executed. An example of a phenotype definition realised in an executable language (Python) is given in Figure 2.

In contrast, the syntax of higher-level modelling languages, while still precise, is often clearer, as well as often being domain specific. For example PhEMA's PhAT allows users to define phenotypes using the high-level, domain-specific syntax

```
valueset={}
valueset[‘‘Acute Pharyngitis”] = ‘‘2.16.840...1011”
valueset[‘‘Acute Tonsilitis”] = ‘‘2.16.840...1012”

def Pharyngitis():
    conditionA = valueset[‘‘Acute Pharyngitis”]
    conditionB = valueset[‘‘Acute Tonsilitis”]
    return conditionA + ‘‘ ” + conditionB;
```

```
valueset ‘‘Acute Pharyngitis”: ‘‘2.16.840...1011”
valueset ‘‘Acute Tonsilitis: ‘‘2.16.840...1012”

define Pharyngitis:
    [Condition: ‘‘Acute Pharyngitis”] union
        [Condition: ‘‘Acute Tonsilitis”]
```

Figure 2. Python (executable) vs. CQL (modelling) [21] representation of Pharyngitis phenotype.

associated with the Quality Data Model's (QDM) logic expressions (now capable of working instead with the Clinical Quality Language (CQL) [7]). Both QDM and CQL make particular provision for the representation of temporal information, such as the (sequential) relationship between events, or between events and defined measurement periods. A further example of a modelling language is OHDSI's cohort definition syntax, which although tied directly to the OMOP CDM, is also high-level and domain specific, allowing for significant clarity when interpreting existing definitions [8]. Like QDM/CQL, this syntax also makes provision for temporal elements (e.g. associating patient observations to an elapsed time period), but looks more holistically at the cohort relating to the phenotype being defined, through, for example, the use of specified inclusion and exclusion criteria. As a final example, *Phenoflow*'s workflow-based model relies on a categorised set of steps to express phenotype definitions, with the same benefits [14]. An example of a phenotype realised in a higher level modelling language (CQL) is also given in Figure 2 for comparison.

It is also important to note that the use of a modelling language as the basis for a phenotype model does not preclude the utility or use of higher-level, (more) human-readable representations such as flowcharts. In fact, modelling languages typically connect well with such representations. For example, flowcharts can be directly generated from Phenoflow's workflow model, QDM is linked to a graphical HTML layer and OHDSI cohorts can be viewed graphically using the *ATLAS* cohort editor.

Support Natural Language Processing-based and Machine Learning-based definitions

The modelling language selected to form the basis of a phenotype definition model should also support the representation of a wider range of definition types (Table 1). That is, under a definition model, one should be able to express not only standard rule-based definitions, but also more complex definitions based on Machine Learning (ML) and Natural Language Processing (NLP) techniques. These techniques are becoming increasingly prevalent, particularly in those situations where the datasets against which the implemented definition is to be executed are of varying completeness or lack consistent record coding. The use of modelling languages to represent these types of definitions is also important for reproducibility, as the use of an abstract representation reduces the potential for references to implemented libraries, commonly used by NLP and ML techniques.

Critically, in order to sufficiently represent both ML and NLP-based phenotypes, a modelling language must be able to represent not only static information (as in rule-based phenotypes) but also complex processes. For example, in the case of ML, a definition may consist of a static, high-level specification of a trained patient classifier (via the provision of values such as feature coefficients), or may be a more complex description of the workflow used to train a classifier for a given condition, with a view to the classifier being re-implemented in new use cases, or training a new model in new use cases, respectively. The workflow used to train a classifier may involve the identification of cases using the presence of certain keywords within an EHR [22] or, as in the case of the *PheNorm* framework, may involve additional steps, such as *normalisation* (to factor in number of encounters when looking at the significance of a larger number of keywords or codes) and *denoising* (to look at the wider context of a keyword or code count, e.g. competing diagnoses) [23]. The high-level definition of machine learning-based, or probabilistic, phenotypes in this way is supported in the OHDSI's Automated PHenotype Routine for Observational Definition, Identification, Training and Evaluation (APHRODITE) computable phenotype framework, which, although also linked to the OMOP CDM, offers a level of abstraction at which trained classifiers can be represented and ported between sites, or a defined workflow can be used to construct site-specific classifiers, when used in an executable form [24].

In the case of NLP, a definition may consist of a simple list of keywords relating to a given medical concept, or a set of regular expressions (not tied to any specific programming language), with a view to these being used as the basis for identifying conditions from free-text in a medical record, when realised in a computable form. However, like ML, NLP-based phenotype definitions are also often associated with complex processes, especially when used to conduct *high-throughput* phenotyping. For example, a *PheMap* phenotype definition consists of a set of linked concepts, the presence of which in a patient's EHR are used to determine the probability of them having the condition represented [25]. The association of a phenotype with different concepts is defined within the PheMap knowledge base, which is constructed based on a process that uses a specific set of NLP tools to derive these associations based on the content of various text-based resources. Therefore, it is also important to represent this process as a part of any definition, especially if the knowledge base needs to be reconstructed within different domains. In instances such as these, it may be important for the definition model used to include guidance on the use of specific tools, but it must do so in a manner that retains clarity and generalisability, thus balancing reproducibility requirements. Modelling languages like CQL have the potential to link to *external tooling*, for the purposes of effectively capturing NLP and ML processes such as these.

Support multi-dimensional descriptions

A significant hurdle in porting a phenotype definition from one setting (institution or dataset) to another – a process we refer to as localisation – is understanding its structure and semantics in order to derive a local computable form, or modify an existing one. Complex rules and the use of idiomatic clinical terminology, although often necessary components of a definition, are both barriers to this understanding, and thus reproducibility. To address this issue, a phenotype definition model should allow an author to express the same logic of a phenotype at different levels of technical complexity. This approach aims to communicate supplementary information alongside the provision of the core definition logic. For example, the workflow-based Phenoflow model allows an author to use the technical terminology and rules required to express a phenotype definition, but then also requires an author to provide longer definitions of this functionality to improve clarity, and to also classify each unit of functionality under a given ontology, enabling a high-level understanding of the functionality to always be accessible. In other modelling languages like CQL, such information can be communicated using constructs such as inline comments.

Logging

The development of a phenotype definition is an incremental process. Capturing and communicating this process is key in ensuring a definition can be accurately interpreted and is thus reproducible. Moreover, this information strengthens the trustworthiness of a phenotype and thus its potential applications. Therefore, phenotype libraries should provide a mechanism for logging the evolution of a phenotype definition.

Support versioning and data provenance

One way in which a phenotype can evolve is through a series of iterative refinements. SAIL Databank's Concept Library stores

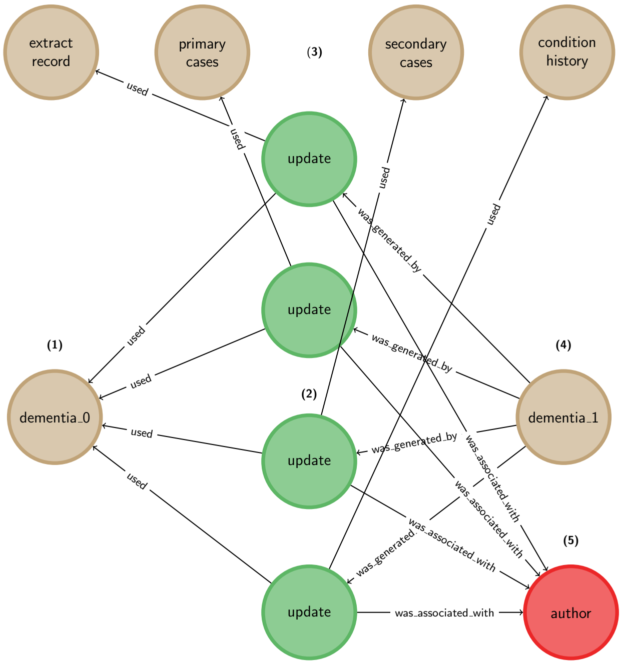

Figure 3. An example data provenance trace showing an update to a dementia phenotype, using the W3C PROV standard. The initial version of the phenotype (1) is updated by four edit activities (2), each of which modifies a component of the definition (e.g. record extract logic, diagnostic codes, previous history) (3), in order to generate a new version (4), and the process is linked with the author making these edits (5).

phenotypes as sets of codes, with a view to making these phenotypes available in different studies and use cases [15]. The concept library, as the name suggests, focuses on a model under which phenotypes are collections of grouped medical *concepts* or *working sets*. The Concept Library records and communicates the evolution of a phenotype definition using methods akin to standard version control, logging the state of a phenotype after each revision, and thus provides an overview of the definition's progression. This versioning process often relies on attributing a universally unique identifier (UUID) to each definition, and each subsequent revision of that definition. Such an identifier might simply be incremental, or convey some details of the phenotype itself. It should also be independent of other identifiers, in order to maximise clarity [26]. For example, within APHRODITE a UUID is derived by committing (each version of) a generated definition to a GitHub repository and extracting the unique commit hash value, in accordance with the FAIR (Findable, Accessible, Interoperable and Reusable) principles [27].

A more comprehensive way to capture the evolution of a definition – and thus contribute to its reproducibility – is to deploy formal *data provenance capture* tools to capture richer, real-time information about the evolution of an entity. This might include information about updates to the structure of a definition, or details of how that definition was validated. It might also include information about how the definition was derived if, for example, the definition is a trained model. An example of one such tool is the *data provenance template server* [28], which allows for the specification of abstract templates, based on the *W3C PROV* standard [29], while eliminating the complexity of dealing with low-level provenance constructs.

Using provenance tools, a trace is automatically constructed that can be *queried* in order to answer a range of questions, such as which clinical codes were used to support a definition at a given time. The Phenoflow library is integrated with the provenance template server, enabling the evolution of the definitions it hosts to be tracked over time [30]. A fragment of provenance constructed in this manner is shown in Figure 3.

Support modular relationships between phenotypes

Another way in which phenotype definitions evolve is through their reuse in constructing new definitions. For example, a phenotype may, either in part or entirely, be defined by other self-contained phenotypes. For example, bipolar disorder is (in part) defined by both substance and alcohol abuse, two phenotypes in their own right [31]. In this way, existing phenotypes become the building blocks for new phenotypes. Much like a version history, it is thus important to capture and communicate this information upon implementation, to provide detailed insight into the formulation of the definition. As such, a phenotype library should log the relationship between different definitions and, if authoring capabilities are supported, a library should allow new definitions to be constructed based upon existing ones. This is similar to the approach taken by the Concept Library, which relates concepts to each other in order to create phenotype definitions, and by Finngen's *Risteys* platform, which relates phenotypes temporally, listing those phenotypes that a patient is likely to exhibit either before or after exhibiting another (e.g. the onset of depression after exhibiting bipolar disorder) [32]. Establishing this relationship further contributes to the provenance of a phenotype, the precision of its definition, and, consequently, its reproducibility.

Conversely, *sub-phenotypes* may be computationally derived from existing phenotypes by clustering of those features (e.g. demographic, diagnosis, medication, etc.) identified, by a trained classifier, to be key attributes of those patients exhibiting the parent phenotype [33]. Such a relationship should also be logged by a phenotype library, to establish the evolution of a definition, and track changes and dependencies across phenotype definitions.

Implementation

Our initial desiderata determined that phenotype definitions should not themselves be executable. While important for reproducibility, this raises natural issues around the complexity of realising a phenotype defined using a modelling language computationally for individual use cases, something that negatively impacts portability. This issue can be addressed by meeting several requirements, which are explored in the following sections.

Communicate implementation information in the model

One way in which implementation can be supported is through the definition itself, by communicating information pertinent to its computable realisation. To do this, one might select a phenotype definition model based on a modelling language that allows an author to express additional information at different levels of *abstraction*. For example, the Phenoflow model frames the traditional (rule-based) logic of a phenotype definition as an *abstract* layer, and allows an author to complement this layer with additional layers, each of which gradually communicates more implementation information: a *functional* layer, introducing the concept of data types, and a *computational* layer, expressing details such as target execution environments. The fact that these layers sit alongside the traditional, abstract logic layer, allows for more concrete implementation to be expressed without impacting portability.

The abstract layer of the Phenoflow model is split into individual modules, each of which represents a distinct unit of functionality, and which collectively define the process required for

deriving a patient cohort from a set of health records. Each module in the abstract layer has an equivalent module in both the functional and computational layers, ensuring a correspondence between each level of representation within the model. However, these modules also provide another means by which implementation information can be communicated through a definition model, in that they provide a clear template for development; each module represents a single unit of functionality that must be implemented by a developer when realising the computable form. This reduces the implementation burden on developers, and thus improves portability. Modelling languages like CQL, which support the definition of individual functions as a part of an abstract layer, offer similar benefits.

Support tooling that provides multiple programming language implementations

Phenotype *implementation tooling* automatically takes an abstract phenotype definition and translates it into a computable form. This naturally improves portability. Examples of this tooling include the *translators* developed by the PhEMA initiative, which are able to take a modelling language definition of a phenotype – such as definitions expressed in QDM, as produced by the PhAT, or in CQL – and transform them into executable formats (e.g pipelines [10]). In addition, the OHDSI tools provide ways to take their domain specific representation and translate it to SQL queries that execute against multiple database systems adopting the OMOP CDM. Although all definitions are SQL, the different dialects used by database vendors are akin to separate programming languages.

Given these benefits, a phenotype library should provide access to implementation tooling. In the simplest form, access should be provided to this tooling by hosting and indexing it in a library, in the same way that the definitions themselves are hosted and indexed. This tooling can then be downloaded, along with a definition, and executed locally in order to produce a computable form. More advanced integrations will provide the functionality offered by implementation tooling directly through the library, by running it as a service that can be accessed by users via the library in order to download the automatically generated computable form of a phenotype. This is the approach taken by the Phenoflow platform, which allows users to obtain computable copies of a phenotype definition directly, by running a microservice generation architecture.

The tooling indexed should be able to support implementations in a variety of different programming languages. While the programming language used might seem to be of little consequence, in practice, even with this presence of a translator, the researcher generating a computable form for a new use case is likely to still have to modify (localise) that computable phenotype for local use. Such modifications might include optimisations to the structure of the implementation to allow the computable form to operate in low-memory environments or to operate as a part of existing infrastructure (e.g. a clinical trial platform [34]). In this instance, having that definition in a language that the researcher is comfortable with editing is important. For example, the pipeline-based implementation originally produced by the PheMA translator only supports the KNIME format. As such, a researcher has to be comfortable with this format in order to make edits. To maximise portability, phenotype libraries should aim to support implementation tooling capable of producing executable definitions in multiple languages. An example of this is seen within the Phenoflow platform, where one can generate a workflow that utilises modules from a variety of languages, including Python and Javascript, with containerised environments supporting the straightforward execution of these units locally.

Support tooling that provides connectivity with multiple data standards

When a phenotype definition is translated by a piece of tooling into an executable form, it is typical for that definition to be tied to a given data source format, from which the resulting cohort is identified. In certain cases, that data format is always the same. For example, OMOP cohort definitions, when translated into a computable form (SQL), are always tailored for the OMOP CDM. While beneficial in the sense that this provides an automated translation process that works across sites, those sites must all adopt the OMOP CDM, which is not always feasible. Instead, in reality, sites may use a variety of implementation formats, such as i2b2 and FHIR. For these reasons, phenotype libraries should index implementation tooling that not only supports multiple language implementations, but also supports the realisation of definitions for different data formats. Naturally, the more data source formats supported, the more portable the definitions stored within a library are. For example, the computable forms generated by PhEMA's translators can be tailored for a variety of local data formats, including FHIR and the OMOP CDM itself. Similarly, in the Phenoflow library, interacting with a data source is considered to be the first step in a phenotype's definition, and as such different *connectors* are available when generating the computable form of a definition. These connectors support a variety of different standards such as OMOP and i2b2, and plans are in place to support dataset specific standards, such as the standard used by UK Biobank (via tooling such as *Funpack* [35]).

The connector approach also provides a natural point at which to conduct any necessary (automatic) translation between the coding system adopted by a target data source, and the coding system expected by the implemented definition. For example, if the target datasource adopts Read codes, but the computable phenotype relies on sets of ICD codes, a connector might not only ingest data, but also perform code mappings accordingly.

Despite these benefits, the requirement to produce a new translator, or new connector, for each new data source format, is a natural drawback to each of these approaches. However, the advantages over manual translation are still clear.

Validation

Validating a phenotype definition involves confirming its accuracy. To do this, the cohort identified by a computable phenotype is typically compared to a *reference standard*, such as the cohort identified by manual chart review from the same patient population (a *gold standard*). The extent to which the two cohorts overlap determines the validity of the definition. While reference standards are a common means of phenotype validation, other techniques exist, and are listed in Table 2. Phenotype definitions that are shown to be accurate are considered to be of a higher quality. Therefore, phenotype libraries should facilitate the validation process.

Support a defined validation process

To support the validation of stored definitions, a phenotype library should have a clear and scalable process for the submission of existing validation information by a user, across a variety of the mechanisms shown in Table 2. This information can then be stored and presented alongside each definition. For example, the CALIBER library stores phenotypes as code sets (342, at the time of writing), with a view to providing a framework for the definition of consistent phenotypes, which can then be reused by care service providers for nationwide EHR-based observational research [13]. Each definition in CALIBER appears alongside algorithmic information about the

Table 2. Phenotype validation mechanisms

Mechanism	Description	Example
Disease registries	Compare the phenotype cohort with those present in the registry.	Comparison of a diabetes phenotype cohort with those patients present in a diabetes registry (e.g. T1D exchange).
Chart review	Compare the phenotype cohort with the patients identified by manual chart review.	Comparison with a diabetes gold standard, produced by double manual chart review of patients.
Cross-EHR concordance	Compare percentage of cases identified by a phenotype across different sources, and identify any overlap.	Comparison of the percentage of patients identified by a diabetes phenotype in primary and secondary care EHRs, and the identification of any case overlap.
Risk factors	Compare the magnitude of the phenotype cohort with standard risk calculations.	Comparison with the output of a Cox hazards model.
Prognosis	Compare the magnitude of the phenotype cohort with external prognosis models.	Comparison with a survival analysis.
Genetic associations	Compare whether the presence of a patient in a phenotype cohort is consistent with their genetic profile.	A patient is more likely to be a valid member of a diabetes cohort if they have the HLA-DR3 gene.

relationship between the code sets and key validation information. Specifically, the CALIBER library offers up to 6 different techniques, which are used to validate a single definition. Similarly, the OHDSI gold standard phenotype library is so-called because there is a well-defined process proposed for the submission of phenotypes based on different user roles. Specifically, the submission of a computable phenotype definition to the library (which can occur using the APHRODITE framework) will require definitions to be submitted by those in the *author* role, vetted by *librarians*, validated by users who act as *validators* and used by standard *users* [36].

Automate multiple validation techniques

When new definitions are submitted without validation information to a library, it should seek to automatically validate these definitions by comparing them, or their outputs, against assets that are hosted alongside the definitions, such as gold standard datasets. For example, in [11], the authors present *electronic phenotyping validation*, a framework for the automated comparison of a definition with manual chart review results. In the absence of such assets, a portal might host tooling designed to derive these assets automatically. For example, *PheValuator* trains a linear model based upon cases and controls identified using some of the techniques already discussed, such as the presence (or absence) of a large number of clinical codes relating to a certain condition within a patient's record [37]. This model is used, in turn, to construct an evaluation cohort, which matches each individual in the cohort to a probability value indicating the likelihood of them having the condition of interest. This cohort can then act as a *silver* standard, against which phenotype definitions can be validated, in this case by using the matched probabilities to construct totals from which sensitivity, specificity and positive predictive value (PPV) are calculated.

There is also an argument for the automated combination of different validation approaches, to avoid the shortcomings of each individual approach. For example, using a disease registry approach alone as a gold standard for phenotypes related to that disease, is not scalable or feasible for patient cohorts focusing on multi-morbidities and complex demographic criteria. Similarly, validating using clinical notes review, where phenotype patient matches are manually reviewed, are not sustainable for large LHS infrastructures. While the manual text extraction of phenotypes can be effective in smaller scenarios, it is heavily dependent on the human expert and the sample being analysed, and not well-suited to cross-site studies with differences in clinical and operational procedures and opinion between sites. As such, phenotype libraries should offer novel hybrid approaches to validation that encompass structured data, free text, and ancillary sources for both structured and unstructured data.

Enable feedback

To facilitate any (informal) user-based validation of stored definitions, a phenotype library should support social interactions between the authors and researchers that use it, with a view to providing authors with feedback and allowing them to address this feedback accordingly. Social functionality is supported by the Phenotype Knowledge Base (PheKB), which currently hosts around 70 phenotype definitions [12]. For example, within the library, users are able to post comments or questions against different phenotypes. A researcher can also request collaboration on the development of phenotype definitions.

However, those users permitted to interact with a phenotype definition within a portal may be restricted. Within PheKB, only users with certain organisational affiliations (e.g. the eMERGE nerwork or the Phenome-Wide Association Studies (PheWAS) community [38]) are provided with access by default, with other users required to request an account prior to providing feedback on definitions. Other portals may restrict access to different countries or regions.

In many cases, these restrictions are necessary during the development of a phenotype. For example, APHRODITE's definition repositories are kept private while they are still under development. However, once developed, definitions can be accessed through the repository via any web browser or through an R Shiny app. Based on practices such as these, phenotype libraries should limit the restrictions they place on those who can engage with the definitions in phenotype libraries, once developed. By eliciting comments on the validity of hosted definitions from a wider audience, one is likely to gain a greater understanding as to the quality of a definition.

Sharing and Warehousing

Once a phenotype definition is appropriately reproducible, portable and validated, it should then be accessible for use by others. While the traditional and default role of a phenotype library is to provide such access, this can be optimised, as discussed in the following sections.

Expose a standard API

To maximise accessibility, a phenotype library should facilitate user interactions via multiple interfaces. The definitions in a library are usually available via a single interface: a graphical front-end. While this provides a reasonable baseline for accessibility, it does not maximise it. For example, a user cannot

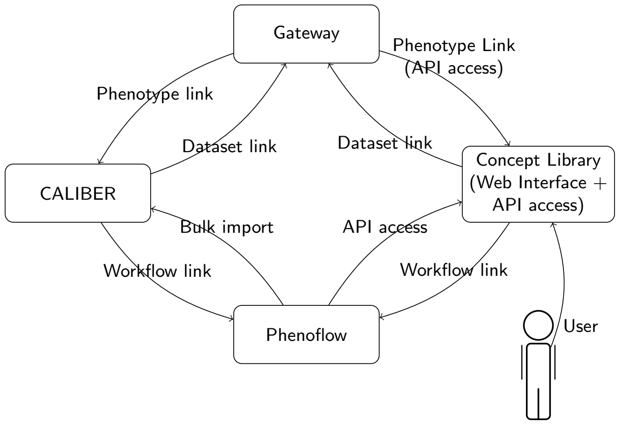

Figure 4. Overview of the services that constitute the HDR UK phenotype library

instruct a piece of software to interact with the library, to include definitions directly within a piece of code, resulting in potential inconsistencies arising from manual entry. Similarly, existing software systems, such as decision-support systems, cannot autonomously access phenotypic information. Perhaps most importantly, a lack of programmatic accessibility means that one library cannot easily access the functionality of another in order to provide complimentary functionality.

To address these issue, phenotype libraries should offer API-level web services that (at a minimum) duplicate the functionality available in a user interface. In doing so, several considerations should be made. Firstly, the level of API access needs to be considered, including whether to provide access only to trusted *partners*, and thus provide suitable authentication mechanisms (e.g. OAuth), or whether to make the API publicly accessible. The selection of the type of API level access provided to the functionality of the web resource should be subject to the policy of the organisation developing the library. Secondly, the protocol used to facilitate communication with the API should be considered, such as Remote Procedure Call (RPC), Service Object Access Protocol (SOAP) and Representation State Transfer (REST). REST is a simple and widely adopted specification model [39], and is thus the technology that is likely to be most attractive when constructing a library API. Next, to support programmatic access and enable definitions to be differentiated automatically, a formal identification system should be established for each definition. The most straightforward way to this is to leverage the UUID attributed to each phenotype version.

The functionality of the API itself also needs to be considered. In [1], the authors propose that an API service should be used to construct phenotype definitions for the purpose of defining inclusion and exclusion criteria for clinical research trials. Building on this outline, we consider several additional API level use cases, including: searching phenotype definitions, extracting a specific phenotype definition, submitting a new phenotype definition, submitting a new use case for an existing phenotype definition or validating an existing definition and linking a phenotype definition with a data source, and vice-versa. Examples of specific functionality that an API level phenomics resource should support within each of these use cases is given in Table 3.

The benefits of API functionality are evident in the CALIBER, Phenoflow and Concept Library libraries, each of which communicate together to collectively form, along with a dataset Gateway, the HDR UK phenomics resource. As shown in Figure 4, each library operates as service, and collectively these services are able to deliver the functionality of a single library to a user. The services at the core of this library are the Concept Library and the CALIBER library, each of which store phenotype definitions. Using provided APIs, the Concept Library is able to import definitions from the CALIBER library, enabling phenotypes to be both formally stored and validated across both services, respectively. Similarly, the Phenoflow service – also capable of automatically importing and representing definitions using a workflow-based model, and generating a corresponding computable form for execution against a local dataset – is able to import definitions from both the Concept Library and CALIBER. Finally, the Gateway service provides access to a comprehensive collection of datasets, which are linked to by services such as CALIBER, when a given phenotype definition is present in one of the hosted datasets. Similarly, the Gateway links back to CALIBER when a phenotype is present in a dataset, in order to facilitate searches based upon these definitions.

Offer advanced search capabilities

The accessibility of existing phenotypes within a library relies on its search capabilities. Searches based on given name or identifier and version should enable simple use cases. For example, PheKB offers comprehensive search functionality, with users not only able to perform searches against the definitions themselves using given keywords, but also against supporting definition content, such as articles, implementations and datasets. Alternatively, the library has the option to list all phenotype definitions – including phenotype definitions under development, if the user is logged in – where a user can instead filter the definitions returned after the fact, based on properties such as the authoring institution.

While the search functionality offered by PheKB is helpful, more advanced search capabilities should be supported to facilitate both more complex cases and improved information retrieval. This includes searches based on specific codes, or groups of codes, or an approximate pattern matching, based on regular patterns or even text similarity. Synonyms (including abbreviations and acronyms) may also be used as a mechanism to improve search results over keyword searches. For example, a search for 'diabetes' would likely fail to find a phenotype that refers to 'T2DM' throughout, although 'T2DM' is a recognised abbreviation that can be semantically linked via the UMLS.

Even more advanced capabilities might include searches employing semantic similarity between a given set of concepts and the stored phenotypes supported by phenotype ontologies [40]. This could enable the discovery of semantically identical or closely related concepts within the library. Similarly, similarity metrics between phenotype definitions, facilitated by the adoption of a formal phenotype model, are likely assist in scalable searches across different repositories, whereby a partial match may indicate a usable cohort definition to investigate.

Include comprehensive metadata

The search and browse features described must be supported by appropriate metadata, which can be used to describe both the subject and format of phenotypes in ways that make them findable to users with specific research or clinical needs. Such definitions we might refer to as 'FAIR Phenotypes' [41]. To achieve this, each phenotype definition should include structured data that describes the subject (i.e., clinical condition) and intent (screening, etc.) of the definition, as well as the source, date, publisher, etc., similar to the tagging of resources in traditional libraries. Additionally, each component of the phenotype model (e.g., underlying data model, data elements, value sets, code lists, coding language) must be specified with an assigned code or value so that users can search on these features or have them displayed when browsing a phenotype

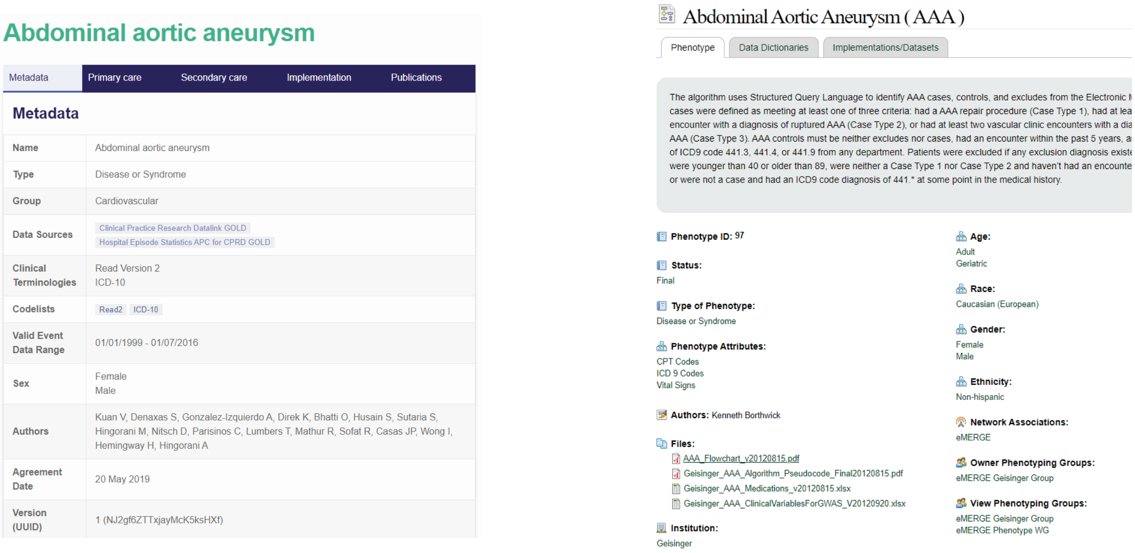

Figure 5. Metadata structure adopted by CALIBER (left) and PheKB (right).

library or repository. Examples of existing libraries that look to attribute appropriate metadata to stored definitions include CALIBER and PheKB (Figure 5).

In addition to supporting search, the use of metadata is important for a number of other reasons. Firstly, metadata can make clear characteristics of phenotypes related to their accessibility, interoperability and re-use. To this end, as part of the Mobilizing Computable Biomedical Knowledge (MCBK) initiative, Alper and Flynn et al. have proposed 12 categories of metadata that are required to fully represent knowledge objects, including phenotypes, for FAIR principled criteria [42]. In addition, metadata fields that describe the versioning aspects of a definition can be populated to further formalise the provenance of the phenotypes in a collection. Next, as the intent, development, and validation of phenotypes are essential for potential implementers to understand in order to trust the quality and appropriateness of a phenotype for a new purpose, representing aspects of the pheontype development and validation process formally is critical. To do this, the Trust and Policy Work Group of the Patient-Centered Clinical Decision Support Learning Network defined an extensive set of metadata for trust [43]. Finally, metadata can be used to formally represent many aspects of the implementation and tooling described, enabling potential implementers to search on these features, such as language, and possibly support automated translations.

While more and robust metadata are beneficial from a library perspective, populating these metadata accurately and consistently requires resources, and the extent and detail of metadata will depend upon a balance to adequately meet the needs at the expense that the library sponsor will bear. One potential solution to this issue is to automatically generate metadata, which is the approach taken in data management platforms [44]. Overall, time will show how the community of phenotype users can develop consensus on a minimum set of metadata, library or indexing best practices to complement and formalise the desiderata described here, and also build a compelling value case for their use to support high quality phenotyping across countries.

Conclusions

While making significant advances, computable phenotyping is still at an early stage where methods and repositories are emerging to meet the needs of a range of medical research domains, with little methodological consensus. As tooling gradually matures beyond the realm of early adopters to become usable for a broad spectrum of researchers and implementers, the focus needs to move away from one-size-fits-all 'perfect' phenotype definitions to acknowledging the diversity of phenotype application areas, the resultant explosion in the numbers and variations of phenotypes to be stored – in particular the arrival of advanced probabilistic and NLP-based phenotypes to sit alongside traditional rule-based definitions – and the challenges of deploying them in the real world, especially in the presence of high-throughput requirements. Portability and reproducibility are essential in addressing this scaling-up, with techniques needed to move phenotype definitions between both data sources and different health settings.

Phenotype libraries offer a natural meeting point of these multiple use-cases and domains to support high-quality phenotype definitions. In terms of designing phenotype libraries as technical entities that enable the storage and retrieval of definitions, there is a clear need to track the evolution of phenotype definitions as they are authored, support advanced search techniques that enable these definitions to be located by others, and establish a collaborative process through which the validity of definitions can be critiqued. All of this functionality should be accessible within a library via multiple channels, in particular comprehensive, standards-based API functionality to ensure interoperability. Authoring and storing phenotype definitions according to a standard model is another aspect through which phenotype libraries can contribute to definition reproducibility. The model adopted by a phenotype library should exist at the correct level of abstraction, prioritising modelling languages over executable programming languages, and offset this, in terms of implementation, by incorporating key implementation information, and improving clarity through multidimensional descriptions. Finally, a phenotype library should encourage the use of phenotype definitions in new use cases by supporting the validation process, both automatically, and through the definition of a structured validation process.

The impact of supporting the development and implementa-

tion of high-quality phenotype definitions is significant, particularly as these definitions provide efficient access to accurate cohort data by overcoming many of the complexities associated with patient datasets. Cohort data not only supports research studies (e.g. the identification of predictors for a certain condition), but also the provision of decision support (e.g. access to the medical histories of one or more individuals) and clinical trials (e.g. the establishment of trial cohorts). The use of computable phenotypes to determine cohorts from complex datasets for these purposes can be complemented by using traditional big data techniques to manage scale; by an increased focus on multi-morbidities – the complex interactions of diseases in patients – which are a crucial factor in personalised decision support systems; and by *N-of-1* clinical trial design.

Overall, running through these desiderata is the awareness that cross-domain sharing of phenotype definitions can only occur through curated libraries that evolve in a controlled manner. Such libraries have to be 1) clinically and scientifically valid; 2) technically realisable; and 3) usable by researchers in different domains. Through the usage of our desiderata, we believe the current and future phenotype libraries will deliver on these three fronts.

Declarations

List of abbreviations

Automated PHenotype Routine for Observational Definition, Identification, Training and Evaluation (APHRODITE); Clinical Quality Language (CQL); Electronic Health Record (EHR); Mobilizing Computable Biomedical Knowledge (MCBK); Observational Health Data Sciences and Informatics (OHDSI); PhEMA Authoring Tool (PhAT); Phenotype Knowledge Base (PheKB); Phenotyping pipeline (PheP); Phenome-Wide Association Studies (PheWAS); Phenotype Execution and Modelling Architecture (PhEMA); Quality Data Model (QDM).

Consent for publication

Not applicable.

Competing Interests

The author(s) declare that they have no competing interests.

Funding

This work was supported by Health Data Research UK, which receives its funding from Health Data Research UK Ltd (NIWA1; GVG and AK: HDRUK/CFC/01) funded by the UK Medical Research Council (MRC), Engineering and Physical Sciences Research Council, Economic and Social Research Council, Department of Health and Social Care (England), Chief Scientist Office of the Scottish Government Health and Social Care Directorates, Health and Social Care Research and Development Division (Welsh Government), Public Health Agency (Northern Ireland), British Heart Foundation, and the Wellcome Trust. In addtion, SD acknowledges that this study is part of the BigData@Heart programme that has received funding from the Innovative Medicines Initiative 2 Joint Undertaking (116074), which receives support from the European Union's Horizon 2020 research and innovation programme (H2020) and the European Federation of Pharmaceutical Industries and Associations (EFPIA). MC and VC are supported by the National Institute for Health Research (NIHR) Biomedical Research Centre based at Guy's and St Thomas' National Health Service Foundation Trust and King's College London, and the Public Health and Multimorbidity Theme of the National Institute for Health Research's Applied Research Collaboration (ARC) South London. GVG and AK also acknowledge support from the NIHR Birmingham Experimental Cancer Medicine Centre (ECMC), the NIHR Birmingham Surgical Reconstruction Microbiology Research Centre (SRMRC) and the NIHR Birmingham Biomedical Research Centre, as well as Nanocommons H2020 (731032) and an MRC fellowship grant (MR/S003991/1). HP acknowledges support from European Molecular Biology Laboratory (EMBL) core funds. LVR and JAP acknowledge support from the National Institute of General Medical Sciences (R01GM105688) and the National Human Genome Research Institute (U01HG011169).

The opinions in this paper are those of the authors and do not necessarily reflect the opinions of the funders.

Authors' Contributions

MC: conceptualisation, methodology, investigation, writing – original draft; SM: conceptualisation, methodology, investigation, writing – original draft; LVR: methodology, writing – original draft; AK: investigation, writing – original draft; GVG: investigation, writing – original draft; CG: investigation, writing – review & editing; DT: investigation, writing – review & editing; JAP: methodology, writing – review & editing; HP: writing – review & editing; RR: investigation, writing – original draft, writing – review & editing; EJ: funding acquisition, writing – review & editing; SD: methodology, funding acquisition, writing – review & editing; VC: funding acquisition, writing – original draft, writing – review & editing;

Acknowledgements

The authors wish to acknowledge the involvement of Dr Susheel Varma (HDR UK) and Dr Elliot Fairweather (King's College London) in the development of this work.

References

1. Richesson R, Smerek M, Cameron CB. A Framework to Support the Sharing and Re-Use of Computable Phenotype Definitions Across Health Care Delivery and Clinical Research Applications. eGEMs (Generating Evidence & Methods to improve patient outcomes) 2016;4(3):10–24.
2. Weaver J, Potvien A, Swerdel J, Voss EA, Hester L, Shoaibi A, et al. Best Practices for Creating the Standardized Content of an Entry in the OHDSI Phenotype Library. In: 5th OHDSI Annual Symposium; 2019. p. 46.
3. Cho K, Introduction to the VA Phenomics Library (VAPheLib);. Available at: "https://www.hsrd.research.va.gov/for_researchers/cyber_seminars/archives/3814-notes.pdf", Accessed on: 2021-02-12.
4. Meineke F, Stäubert S, Löbe M, Uciteli A, Löffler M. Design and Concept of the SMITH Phenotyping Pipeline. Studies in health technology and informatics 2019;267:164–172.
5. Clarivate Analytics, Web of science;. Available at: http://www.webofknowledge.com, Accessed 2021-02-15.
6. Pacheco JA, Rasmussen LV, Kiefer RC, Campion TR, Speltz P, Carroll RJ, et al. A case study evaluating the portability of an executable computable phenotype algorithm across multiple institutions and electronic health record environments. Journal of the American Medical Informatics Association 2018;25(11):1540–1546.

7. Brandt PS, Kiefer RC, Pacheco JA, Adekkanattu P, Sholle ET, Ahmad FS, et al. Toward cross-platform electronic health record-driven phenotyping using Clinical Quality Language. Learning Health Systems 2020;4(4):9–17.
8. Hripcsak G, Shang N, Peissig PL, Rasmussen LV, Liu C, Benoit B, et al. Facilitating phenotype transfer using a common data model. Journal of Biomedical Informatics 2019;96:120–127.
9. Banda JM, Halpern Y, Sontag D, Shah NH. Electronic phenotyping with APHRODITE and the Observational Health Sciences and Informatics (OHDSI) data network. In: Proceedings of AMIA Joint Summits on Translational Science; 2017. p. 48–57.
10. Mo H, Pacheco JA, Rasmussen LV, Speltz P, Pathak J, Denny JC, et al. A Prototype for Executable and Portable Electronic Clinical Quality Measures Using the KNIME Analytics Platform. In: Proceedings of AMIA Joint Summits on Translational Science, vol. 2015; 2015. p. 127–31.
11. Kukhareva P, Staes C, Noonan KW, Mueller HL, Warner P, Shields DE, et al. Single-reviewer electronic phenotyping validation in operational settings: Comparison of strategies and recommendations. Journal of Biomedical Informatics 2017;66(C):1–10.
12. Kirby JC, Speltz P, Rasmussen LV, Basford M, Gottesman O, Peissig PL, et al. PheKB: a catalog and workflow for creating electronic phenotype algorithms for transportability. Journal of the American Medical Informatics Association 2016;23(6):1046–1052.
13. Denaxas S, Gonzalez-Izquierdo A, Direk K, Fitzpatrick NK, Fatemifar G, Banerjee A, et al. UK phenomics platform for developing and validating electronic health record phenotypes: CALIBER. Journal of the American Medical Informatics Association 2019;26(12):1545–1559.
14. Chapman M, Rasmussen L, Pacheco J, Curcin V. Phenoflow: A Microservice Architecture for Portable Workflow-based Phenotype Definitions. In: Proceedings of AMIA Joint Summits on Translational Science; 2021. p. 142–151.
15. SAIL Databank, The Concept Library;. Available at: https://conceptlibrary.demo.saildatabank.com/home/, Accessed on: 2021-02-11.
16. Boxwala AA, Rocha BH, Maviglia S, Kashyap V, Meltzer S, Kim J, et al. A multi-layered framework for disseminating knowledge for computer-based decision support. Journal of the American Medical Informatics Association 2011;18(Supplement_1):132–139.
17. Curcin V. Why does human phenomics matter today? Learning Health Systems 2020;4(4):1–3.
18. Sá-Sousa A, Jacinto T, Azevedo LF, Morais-Almeida M, Robalo-Cordeiro C, Bugalho-Almeida A, et al. Operational definitions of asthma in recent epidemiological studies are inconsistent. Clinical and Translational Allergy 2014;4(1):24.
19. Mo H, Thompson WK, Rasmussen LV, Pacheco JA, Jiang G, Kiefer R, et al. Desiderata for computable representations of electronic health records-driven phenotype algorithms. Journal of the American Medical Informatics Association 2015;22(6):1220–1230.
20. Papez V, Denaxas S, Hemingway H. Evaluating OpenEHR for Storing Computable Representations of Electronic Health Record Phenotyping Algorithms. In: Proceedings - IEEE Symposium on Computer-Based Medical Systems; 2017. p. 509–514.
21. Jiang G, Prud'Hommeaux E, Xiao G, Solbrig HR. Developing A Semantic Web-based Framework for Executing the Clinical Quality Language Using FHIR. In: CEUR Workshop Proceedings; 2017. p. 126–130.
22. Agarwal V, Podchiyska T, Banda JM, Goel V, Leung TI, Minty EP, et al. Learning statistical models of phenotypes using noisy labeled training data. Journal of the American Medical Informatics Association 2016;23(6):1166–1173.
23. Yu S, Ma Y, Gronsbell J, Cai T, Ananthakrishnan AN, Gainer VS, et al. Enabling phenotypic big data with PheNorm. Journal of the American Medical Informatics Association 2018;25(1):54–60.
24. Banda JM, Seneviratne M, Hernandez-Boussard T, Shah NH. Advances in electronic phenotyping: from rule-based definitions to machine learning models. Annual review of biomedical data science 2018;1:53–68.
25. Zheng NS, Feng Q, Eric Kerchberger V, Zhao J, Edwards TL, Cox NJ, et al. PheMap: A multi-resource knowledge base for high-throughput phenotyping within electronic health records. Journal of the American Medical Informatics Association 2020;27(11):1675–1687.
26. Cimino JJ. Desiderata for controlled medical vocabularies in the twenty-first century. Methods of Information in Medicine 1998;37(04/05):394–403.
27. Banda JM, Williams A, Kashyap M, Seneviratne MG, Potvien A, Duke J, et al. FAIR Phenotyping with APHRODITE. In: 5th OHDSI Annual Symposium; 2019. p. 45.
28. Curcin V, Fairweather E, Danger R, Corrigan D. Templates as a method for implementing data provenance in decision support systems. Journal of Biomedical Informatics 2017;65:1–21.
29. Moreau L, Missier P, Belhajjame K, B'Far R, Cheney J, Coppens S, et al. PROV-DM: The PROV Data Model. World Wide Web Consortium; 2013.
30. Fairweather E, Chapman M, Curcin V. A delayed instantiation approach to template-driven provenance for electronic health record phenotyping. In: Proceedings of the 9th International Provenance and Annotations Workshop, IPAW 2021 (In press); 2021. .
31. Castro VM, Minnier J, Murphy SN, Kohane I, Churchill SE, Gainer V, et al. Validation of electronic health record phenotyping of bipolar disorder cases and controls. American Journal of Psychiatry 2015;172(4):363–372.
32. FinnGen, Risteys: Explore FinnGen data at the phenotype level;. Available at: https://risteys.finngen.fi/, Accessed 2021-03-05.
33. Xu Z, Wang F, Adekkanattu P, Bose B, Vekaria V, Brandt P, et al. Subphenotyping depression using machine learning and electronic health records. Learning Health Systems 2020;4(4):40–49.
34. Chapman M, Domínguez J, Fairweather E, Delaney BC, Curcin V. Using Computable Phenotypes in Point-of-Care Clinical Trial Recruitment. In: Digital Personalized Health and Medicine - Proceedings of MIE 2021 (In press); 2021. .
35. McCarthy P, funpack. Zenodo; 2021. 10.5281/zenodo.4646309.
36. Knoll C, Banda J, Rao G, Chen R, Swerdel J. OHDSI Gold Standard Phenotype Library. Observational Health Data Sciences and Informatics; 2019.
37. Swerdel JN, Hripcsak G, Ryan PB. PheValuator: Development and evaluation of a phenotype algorithm evaluator. Journal of Biomedical Informatics 2019;97.
38. Denny JC, Ritchie MD, Basford MA, Pulley JM, Bastarache L, Brown-Gentry K, et al. PheWAS: demonstrating the feasibility of a phenome-wide scan to discover gene-disease associations. Bioinformatics 2010;26(9):1205–1210.
39. Fielding RT. Architectural styles and the design of network-based software architectures. PhD thesis, University of California, Irvine; 2000.
40. Gkoutos GV, Schofield PN, Hoehndorf R. The anatomy of phenotype ontologies: principles, properties and applications. Briefings in bioinformatics 2018;19(5):1008–1021.

41. Wilkinson MD, Dumontier M, Aalbersberg IJ, Appleton G, Axton M, Baak A, et al. The FAIR Guiding Principles for scientific data management and stewardship. Scientific Data 2016;3(1):1–9.
42. Alper B, Flynn A, Bray B. Categorizing Metadata to Help Mobilize Computable Biomedical Knowledge. Learning Health Systems (Under Review) 2021;.
43. Richardson JE, Middleton B, Platt JE, Blumenfeld BH. Building and maintaining trust in clinical decision support: Recommendations from the Patient-Centered CDS Learning Network. Learning Health Systems 2020;4(2):7–14.
44. Nind T, Galloway J, McAllister G, Scobbie D, Bonney W, Hall C, et al. The research data management platform (RDMP): A novel, process driven, open-source tool for the management of longitudinal cohorts of clinical data. GigaScience 2018;7(7):1–12.

Table 3. Suggested library API functions. All requests made in, and responses returned in, YAML+Markdown/JSON/XML formats.

Function		User Access Level	Description
Search	Simple Search	Public	A free text search, examining the entire contents of the portal and returning a list of phenotypes that match the search criteria.
	Advanced Search	Public	A free text search, examining specified sections of the portal (e.g., main content, just metadata, etc.) and returning a list of phenotypes that match the search criteria.
Phenotype extraction	Extracting specific phenotype(s)	Public	Given a phenotype ID supplied by a user (or generated by the platform), the API returns the phenotype definition.
	Extracting all phenotypes	Public	Return a full list of phenotypes.
Adding new phenotype(s)		Authorised users	Only authorised users should be allowed to either submit a single or group of phenotype definitions.
Updating a phenotype definition	Updating the contents of a specific phenotype	Authorised users	Each aspect of a phenotype definition – including constituent codelists, links to datasets where that phenotype appears, and other metadata – can be updated by passing a phenotype ID and the names of the fields to update and their new values. Each update should mark a version number to keep record of any updates over time.
	Updating a complete phenotype with multiple features	Authorised users	Update a phenotype contents by passing a phenotype ID and submitting an updated phenotype definition file to replace the previous version for public view.
	Submission of a new validation case study for an existing phenotype	Authorised users	Adding a new use case to validate an existing phenotype (identified by a phenotype ID) by passing a file.
Deletion of a phenotype	Removing a phenotype from public view (soft delete)	Private to portal administrators	An administrator of the portal can hide a phenotype definition by providing a phenotype ID.
	Removing a phenotype from the library (hard delete).	Private to portal administrators	An administrator of the portal can delete a phenotype definition entirely by providing a phenotype ID.
