# Supplementary material for: Desiderata for the development of next-generation electronic health record phenotype libraries
Source: Gigascience. 2021 Sep 11;10(9):giab059. doi: 10.1093/gigascience/giab059 (PMC8434766; doi:10.1093/gigascience/giab059)
Supplement: giab059_Response_to_Reviewer_Comments_Original_Submission [file giab059_response_to_reviewer_comments_original_submission.pdf]

# Author's Response To Reviewer Comments

Close

Department of Population Health Sciences
King's College London
London
SE1 1UL

July 15, 2021

Gigascience - Response to reviews

We would like to thank both reviewers for taking the time to produce excellent and in-depth reviews. Responses to the comments are provided below, many of which correspond to changes in the original article, and are highlighted in the version with tracked changes. We hope that we have been able to adequately address all the concerns.

* Reviewer 1

"In this work, the authors present a set of 13 desiderata to guide the development of future phenotype libraries.
The work presented here nicely rounds out current and established phenotyping efforts and established/outlines their suitability and components for a larger and broader definition of a phenotype library.
The relevant literature is well collected, and with the exception of newer developments (within the last 9 months) for the OHDSI phenotype library (https://data.ohdsi.org/PhenotypeLibrary/) and tools (https://pubmed.ncbi.nlm.nih.gov/31369862/) is highly relevant and up to date.
Each current tool is nicely analyzed and dissected by the authors to deliberate over the items that are included in the desiderata they propose."

> We thank the reviewer for their encouraging feedback, and agree that recent developments in the OHDSI network will indeed be a useful addition. We have now made changes in the text to recognise the initial deployment of the Gold Standard OHDSI Phenotype library (`Background', Page 2, 9th paragraph) and the PheValuator tool (`Automated multiple validation techniques', Page 8, 1st (full) paragraph).

"The figures and tables are well utilized and relevant, but a missing opportunity is a more comprehensive table that includes their 13 elements as columns and the current available libraries/tools as rows, with checkmarks as to which elements they provide in perspective to the 13 provided here."

> We agree that such a table would be useful, but do have some concerns about trying to draw comparisons between different libraries and tools under our desiderata in this manner.
For example, our desiderata focus on broad features and principles, which are often still under development in existing systems or exist in various forms that are not easily aligned.
We hope that our focus in this work will help advance the field to the stage where a meaningful direct comparison, such as the one suggested, can be made between different systems.

"One considerable concern is that the 13 desiderata feel like they are all proposed based on the authors' works (CALIBER and PhenoFlow), serving more of a way to fit these contributions to a broader context, than an impartial discussion about what phenotype libraries would need based on current literature. Some changes in the language would greatly improve this, or the paper focus should be the phenotype library that the authors have built, versus the other approaches - which does not seem to be the way the manuscript is currently presented."

> We agree that a significant number of our desiderata are based upon the functionality offered by the tools and libraries developed within the authors' own phenomics communities.
In this form, the desiderata do indeed operate as `lessons learned', representing practices that have

lead to the development of high-quality phenotype definitions and can thus inform the wider phenomics community.
We have clarified this at various points within the manuscript, including the abstract, introduction (Page 2, 5th paragraph) and methods (Page 3, 1st, 2nd and 3rd paragraphs) sections.

> To ensure that we are reflecting a broader perspective, our desiderata are further informed by our review of the functionality offered by tools outside of the authors' phenomics communities, such as those developed within the OHDSI network.
Thus, we would prefer to retain the concept of desiderata to allow ourselves the flexibility to also make reference to these externally developed tools, but the aforementioned additions to the manuscript make clear that the authors' own work contributes significantly towards the practices put forward.
The use of the term also gives us the flexibility to discuss our vision for future directions, albeit still grounded in concrete experiences.

"Other than this concern, this work is highly relevant and very useful for the communities involved in building phenotyping libraries."

> We thank the reviewer for all their positive remarks.

* Review 2

"High-quality phenotype definitions are desirable for clinical research.
A phenotype library of portable, reproducible and validated phenotyping definitions will be valuable for the research community.
The authors examined the work phenotyping models, implementation and validation, and summarized several desiderata for best practices in this review.
Some points mentioned in the paper were similar to the previous report cited (https://academic.oup.com/jamia/article/22/6/1220/2357938)."

> We thank the reviewer for their in-depth summary of the work.

"My primary concern regarding this piece of work is the phenotyping scope.
The discussion and thoughts fit well for most rule-based phenotype definitions.
However, more and more phenotyping research moves forward to either machine-learning-based or high-throughput approaches (e.g., PheMAP and PheNorm).
Therefore, it is necessary to add discussions on these approaches.
In addition, NLP algorithms could be vastly complicated.
Therefore, it is essential to add more discussions regarding the complexity beyond NLP languages and packages."

> We agree that an increased focus on machine-learning-based and natural language processing-based/high-throughput approaches is required.
We have added additional recognition for these approaches, alongside traditional rule-based approaches, at various points in the article, including our introduction to phenotyping (Page 2, 1st paragraph), our closing discussion (Page 10, 3rd (full) paragraph) and within the desiderata themselves (Page 5, 2nd, 3rd and 4th paragraphs).

> In the latter case, we have developed an additional desideratum within the `models' section -- `Support Natural Language Processing-based and Machine Learning-based definitions' -- which significantly expands upon our comments about the importance of abstract models in representing a wider range of definition types, including ML and NLP approaches.
Specifically, we have expanded our discussion of NLP-based phenotypes, discussing complex processes such as those associated with the derivation of the PheMap knowledge base.
In addition, we have expanded our discussion on machine learning-based approaches, to provide more details on the processes for deriving probabilistic phenotypes, such as the operation of the PheNorm algorithm.

* Editor

"- reviewer 1 points out that ` the 13 desiderata feel like they are all proposed based on the authors' works (CALIBER and PhenoFlow)'. For a narrative review article such as this, it is not a problem if it presents `lessons learned' based on the authors' own work, but I agree with the reviewer that this

should be reflected in the language of the article.
- reviewer 2 feels a section on machine-learning-based and high-throughput approaches is needed."

> We hope that, in our response to the individual reviewers, we have been able to address these concerns.

"In addition, I recommend to improve the title of the article, to make it clear it's about phenotype libraries in a clinical context. GigaScience is a multidisciplinary journal and I think it would be wise to make it clear in the title that this review is about phenotypes in the context of health records."

> We have altered the title accordingly.

Best regards,

Martin Chapman

Close
