# Supplementary material for: Desiderata for the development of next-generation electronic health record phenotype libraries
Source: Gigascience. 2021 Sep 11;10(9):giab059. doi: 10.1093/gigascience/giab059 (PMC8434766; doi:10.1093/gigascience/giab059)
Supplement: giab059_Reviewer_1_Report_Original_Submission — Juan M. Banda, Ph.D -- 6/25/2021 Reviewed [file giab059_reviewer_1_report_original_submission.pdf]

Reviewer Report

Title: Desiderata for the development of next-generation electronic health record phenotype libraries

Version: Original Submission **Date:** 6/25/2021

Reviewer name: Juan M. Banda, Ph.D

Reviewer Comments to Author:

In this work, the authors present a set of 13 desiderata to guide the development of future phenotype libraries. The work presented here nicely rounds out current and established phenotyping efforts and established/outlines their suitability and components for a larger and broader definition of a phenotype library. The relevant literature is well collected, and with the exception of newer developments (within the last 9 months) for the OHDSI phenotype library (https://data.ohdsi.org/PhenotypeLibrary/) and tools (https://pubmed.ncbi.nlm.nih.gov/31369862/) is highly relevant and up to date. Each current tool is nicely analyzed and dissected by the authors to deliberate over the items that are included in the desiderata they propose. The figures and tables are well utilized and relevant, but a missing opportunity is a more comprehensive table that includes their 13 elements as columns and the current available libraries/tools as rows, with checkmarks as to which elements they provide in perspective to the 13 provided here. One considerable concern is that the 13 desiderata feel like they are all proposed based on the authors' works (CALIBER and PhenoFlow), serving more of a way to fit these contributions to a broader context, than an impartial discussion about what phenotype libraries would need based on current literature. Some changes in the language would greatly improve this, or the paper focus should be the phenotype library that the authors have built, versus the other approaches - which does not seem to be the way the manuscript is currently presented. Other than this concern, this work is highly relevant and very useful for the communities involved in building phenotyping libraries.

Methods

Are the methods appropriate to the aims of the study, are they well described, and are necessary controls included? Choose an item.

Conclusions

Are the conclusions adequately supported by the data shown? Choose an item.

Reporting Standards

Does the manuscript adhere to the journal's guidelines on minimum standards of reporting? Choose an item.

Choose an item.

Statistics

Are you able to assess all statistics in the manuscript, including the appropriateness of statistical tests used? Choose an item.

Quality of Written English

Please indicate the quality of language in the manuscript: Choose an item.

Declaration of Competing Interests

Please complete a declaration of competing interests, considering the following questions:

- Have you in the past five years received reimbursements, fees, funding, or salary from an organisation that may in any way gain or lose financially from the publication of this manuscript, either now or in the future?
- Do you hold any stocks or shares in an organisation that may in any way gain or lose financially from the publication of this manuscript, either now or in the future?
- Do you hold or are you currently applying for any patents relating to the content of the manuscript?
- Have you received reimbursements, fees, funding, or salary from an organization that holds or has applied for patents relating to the content of the manuscript?
- Do you have any other financial competing interests?
- Do you have any non-financial competing interests in relation to this paper?

If you can answer no to all of the above, write 'I declare that I have no competing interests' below. If your reply is yes to any, please give details below.

No competing interest

I agree to the open peer review policy of the journal. I understand that my name will be included on my report to the authors and, if the manuscript is accepted for publication, my named report including any attachments I upload will be posted on the website along with the authors' responses. I agree for my report to be made available under an Open Access Creative Commons CC-BY license (http://creativecommons.org/licenses/by/4.0/). I understand that any comments which I do not wish to be included in my named report can be included as confidential comments to the editors, which will not be published.

Choose an item.

To further support our reviewers, we have joined with Publons, where you can gain additional credit to further highlight your hard work (see: https://publons.com/journal/530/gigascience). On publication of this paper, your review will be automatically added to Publons, you can then choose whether or not to claim your Publons credit. I understand this statement.

Yes Choose an item.
